# AI-driven integration of Framingham Heart Study data with machine learning, deep learning, and explainable AI for enhanced pharmaceutical marketing

**DOI:** 10.1038/s41598-026-45770-0

**Published:** 2026-04-09

**Authors:** Meghna Chaudhary, M. Afshar Alam, Sherin Zafar, Kashish Ara Shakil, Mudasir Ahmad Wani, Sulieman Alshuhri, Kiliyanal Muhammedkunju Abubeker

**Affiliations:** 1 Department of CSE, SEST, Jamia Hamdard, New Delhi, India; 2 Department of Computer Sciences, College of Computer and Information Sciences, Princess Nourah bint Abdulrahman University, P.O. Box 84428, Riyadh, 11671 Saudi Arabia; 3 College of Computer and Information Sciences, Imam Mohammad Ibn Saud Islamic University (IMSIU), Riyadh, 13318 Saudi Arabia; 4 Department of Electronics and Communication Engineering, Amal Jyothi College of Engineering (Autonomous), Kanjirappally, Kerala India

**Keywords:** Framingham Heart Study, Machine learning (ML), Deep learning (DL), Explainable artificial intelligence (XAI), Pharmaceutical marketing, Marketing mix modeling, Healthcare data analytics, Predictive analytics, Data-driven decision making, Computational biology and bioinformatics, Health care, Mathematics and computing, Medical research

## Abstract

AI algorithms, in drug discovery, support target identification by recognizing biological patterns and molecular interactions linked to disease mechanisms. They further aid in lead compound optimization, virtual screening of large chemical libraries, and do drug design, thereby reducing time and cost constraints traditionally associated with laboratory-based approaches. This research proposes a novel framework that integrates the Framingham Heart Study (FHS)—a gold standard longitudinal dataset in cardiovascular research—with advanced machine learning (ML), deep learning (DL), and explainable artificial intelligence (XAI) techniques to predict the risk of death or survival probability based on cardiovascular risk factors and to enhance pharmaceutical marketing precision. The study leverages structured data from FHS, encompassing risk factors such as age, cholesterol levels, blood pressure, smoking status, and diabetes incidence, to model predictive relationships that inform patient-specific therapeutic interventions. Using reliability-centric ensemble ML approaches like random forest and XG Boost, alongside DL architectures including feedforward neural networks, the approach uncovers non-linear patterns and latent associations in patient behavior and treatment outcomes. To address the opacity often associated with complex models, XAI methods such as SHAP values and LIME are deployed to render outputs interpretable, thus aligning with medical ethics and regulatory standards. The results and Explainable AI methods, such as SHAP and LIME that are employed to interpret complex model predictions and ensure transparency for stakeholders, demonstrate how the insights can be translated into evidence-based pharmaceutical marketing mix modeling, supporting targeted interventions, efficient market segmentation, and personalized patient engagement strategies. The proposed approach bridges the gap between clinical research and marketing strategy, offering a data-driven pathway to enhance decision-making in the pharmaceutical sector while maintaining patient-centered practices. In this study, multiple machine learning and deep learning models, including support vector machine (SVM), random forest, XGBoost, logistic regression, feed forward neural network (FFNN), and multi-layer perceptron (MLP), were systematically evaluated for predicting cardiovascular disease outcomes using the Framingham Heart Study dataset. The SVM model demonstrated superior performance, achieving the highest test accuracy (96.65%), precision (96.55%), recall (87.50%), F1 score (91.80%), and ROC AUC (99.00%), outperforming all other baseline and deep learning models. In contrast, the FFNN and MLP models exhibited moderate performance, with final test accuracies of approximately 79.88% and 95.98%, respectively. The ensemble base learners, including XGBoost, random forest, and logistic regression, achieved lower accuracies (ranging from 71% to 79%) and reduced recall rates, indicating limitations in correctly identifying high-risk cases. Interpretability through LIME further validated the SVM’s robust decision boundaries and its alignment with clinically significant risk factors. Overall, the comparative results establish the SVM as the most reliable and generalizable predictive model for early cardiovascular risk stratification in the Framingham cohort.

## Introduction

In the modern era of healthcare innovation, the intersection of epidemiological research and artificial intelligence (AI) is reshaping the landscape of pharmaceutical decision-making. With vast biomedical datasets now accessible, the ability to derive actionable insights has become central to both clinical outcomes and commercial strategies. Among the most pivotal resources in cardiovascular epidemiology is the Framingham Heart Study (FHS)—a longitudinal cohort initiated in 1948 that has served as the foundation for numerous predictive models in cardiovascular risk stratification^1^. The comprehensiveness of this dataset, which includes clinical biomarkers, lifestyle factors, genetic information, and longitudinal health outcomes, makes it an invaluable asset for data-driven research.

At the core of this study is the integration of machine learning (ML), deep learning (DL), and explainable artificial intelligence (XAI) techniques to extract meaningful, reliable patterns from the FHS dataset. ML and DL offer powerful tools for identifying latent variables and nonlinear associations in complex medical data, enabling models that can predict patient responses, adherence probabilities, and therapeutic trajectories^2^. However, the inherent opacity of these algorithms poses challenges in terms of trust, regulatory compliance, and ethical transparency—especially in high-stakes environments like pharmaceutical marketing.

To mitigate these issues, XAI frameworks such as SHAP (SHapley Additive ex Planations), LIME (Local Interpretable Model-Agnostic Explanations), and attention mechanisms in neural networks are employed to demystify algorithmic outcomes. These tools enhance model interpretability and support fair decision-making, aligning with regulatory standards^3^. From a marketing standpoint, AI-driven segmentation and profiling derived from epidemiological data empower pharmaceutical stakeholders to tailor outreach strategies based on population-level disease trends, predicted patient behaviors, and treatment efficacy clusters^4^. By adopting reliability-centric evaluation metrics—including precision, calibration, and robustness assessments—this integrative approach ensures that the models not only perform well statistically but also retain clinical and ethical fidelity^5^. The research presented herein underscores the potential of harmonizing longitudinal medical data with advanced computational intelligence to inform ethical, personalized, and impactful pharmaceutical marketing decisions. In doing so, it advocates for a paradigm where commercial healthcare strategies are rooted in scientific rigor and guided by transparent AI^6^.

This paper proposes an integrative approach that bridges epidemiological insights from the FHS with AI-driven modeling techniques to enable pharmaceutical marketers to segment audiences, personalize messaging, and promote responsible therapeutic targeting. In doing so, the research contributes to an emerging paradigm where healthcare analytics inform ethical and impactful commercial strategies.

In a different domain, Marketing Mix Modeling (MMM) applies similar quantitative principles to understand the impact of marketing variables—product, price, place, promotion—on sales or other KPIs. MMM typically uses historical data and regression-based models to isolate the effect of each marketing lever (e.g., advertising spend, discount campaigns, distribution coverage) while controlling for external variables like seasonality, competitor activity, and macroeconomic factors^7^. The goal is to optimize marketing investment by understanding the ROI of each channel or tactic, much like the FHS sought to optimize public health interventions by understanding the effect size of each risk factor. The core philosophical alignment between FHS and MMM lies in their systemic, data-driven approach to causal inference. Both frameworks treat their target outcome (heart disease in FHS; sales or brand metrics in MMM) as a result of multiple interrelated and potentially confounding variables. They aim to derive partial effects—the unique contribution of each factor after controlling for others—and build interpretable models that support both predictive accuracy and decision-making. Just as FHS employs logistic regression or Cox proportional hazards models to estimate disease risk, MMM often relies on linear or nonlinear regression to estimate marketing elasticity, saturation points, and interaction effects.

### Importance and relevance

The integration of artificial intelligence with longitudinal epidemiological datasets represents a transformative shift in both healthcare analytics and pharmaceutical strategy. In this context, the Framingham Heart Study (FHS)—one of the most influential and richly detailed cardiovascular datasets globally—serves as a critical anchor for developing predictive, explainable, and ethically aligned marketing models^8^. Its longitudinal nature allows for robust modelling of disease progression, therapeutic effectiveness, and behavioural health trends across diverse population cohorts. Pharmaceutical marketing, traditionally guided by heuristic approaches and retrospective sales data, now has the opportunity to transition into a more data-driven paradigm^9^. By embedding machine learning (ML) and deep learning (DL) frameworks into the analytical pipeline, this research enables the identification of high-risk patient segments, personalized therapeutic outreach, and evidence-based promotional strategies^10^. Moreover, the deployment of explainable AI (XAI) techniques ensures that these insights are transparent, interpretable, and compliant with regulatory standards—thereby fostering trust among stakeholders, clinicians, and regulators. Pharmaceutical companies are increasingly required to demonstrate accountability in targeting and messaging, particularly when patient privacy, algorithmic bias, and therapeutic equity are at stake^11^. By aligning predictive modelling with epidemiological rigor and XAI, this research provides an approach that enhances both commercial effectiveness and responsibility. Ultimately, this study advances a multidisciplinary agenda—bridging public health, computational intelligence, and marketing strategy—while contributing to the broader discourse on responsible AI in healthcare. Its findings are expected to influence not only marketing outcomes but also policy development, patient empowerment, and the global conversation on equitable access to healthcare innovations.

### Major problems identified

Despite the potential of integrating longitudinal epidemiological data with artificial intelligence (AI) for pharmaceutical marketing, several core challenges hinder optimal implementation. These problems span across data quality, model robustness, interpretability, ethical usage, and strategic alignment, all of which must be addressed for reliable, transparent, and effective outcomes.

- Heterogeneity and complexity of longitudinal medical data.
- Limitations in model generalizability and overfitting risks.
- Clinical misalignment of explainable AI outputs.
- Ethical tensions in data-driven marketing strategies.
- Inadequacy of conventional evaluation metrics.
- Fragmentation between marketing objectives and clinical impact.

### Motivation

The rapid digitization of healthcare systems and the explosion of real-world biomedical data have created a unique opportunity to reimagine pharmaceutical marketing as a science-led, ethically grounded discipline. Traditional marketing approaches—often reliant on broad demographic targeting and retrospective sales analytics—fall short in capturing the nuanced needs, behaviors, and risk profiles of modern patients. There exists a compelling need to shift toward evidence-based strategies that are both clinically informed and technologically intelligent. The Framingham Heart Study (FHS), a landmark in cardiovascular epidemiology, presents an untapped reservoir of longitudinal medical data with rich temporal, clinical, and behavioral attributes. Its depth and historical relevance offer a fertile testing ground for machine learning (ML) and deep learning (DL) models capable of extracting non-obvious patterns in patient trajectories, treatment responsiveness, and disease progression. However, unlocking this potential demands more than just predictive accuracy—it requires trust, interpretability, and actionable relevance. This is where the fusion with explainable artificial intelligence (XAI) becomes critical. In pharmaceutical marketing, decisions must not only be accurate but also transparent, especially when they influence therapeutic messaging, resource allocation, or patient engagement. By embedding XAI into the modelling pipeline, stakeholders gain clarity into the “why” behind recommendations, aligning marketing actions with clinical rationale and ethical practice. The motivation for this research, therefore, stems from a desire to implement a method where epidemiological rigor meets algorithmic precision—and where commercial strategies are reshaped to serve both health outcomes and ethical standards. It envisions a future where marketing is no longer isolated from scientific insight but deeply interconnected with it, creating a more informed, responsible, and impactful healthcare ecosystem. The Framingham Heart Study primarily collects data on cardiovascular disease risk factors and incidence of events like heart attacks, strokes, and other health outcomes. It does include mortality data, but the classic version of the dataset that’s widely used (like the popular Framingham CVD Risk dataset) usually contains variables like age, cholesterol, blood pressure, diabetes, smoking status, but not an explicit “survived/died” label in a simple form.

### Contributions

The key contributions of this paper are summarized as follows:

- Implementation of an AI-integrated techniques for pharmaceutical insights.
- Application of reliability-centric modelling techniques.
- Advancement of context-aware explainability in healthcare AI.
- Strategic use of longitudinal epidemiological data.
- Ethical marketing model incorporating responsible AI principles.
- Empirical validation for therapeutic segmentation and targeting.

### Identifying and addressing technological challenges

- Model selection for accuracy and generalization.
- Handling longitudinal data complexity.
- Enhancing explainability for clinical use.
- Ensuring data privacy and regulatory compliance.
- Integrating reliability-centric evaluation.
- Technical alignment with marketing pipelines.

The format of this research is as follows: “Literature review” section examines relevant research in the area of predicting and estimating survival rates and mortality risk using predictive models trained on Framingham Heart Study data. “Proposed methodologies” section outlines the methods and materials, including marketing mix modelling, explainable artificial intelligence (XAI) techniques, and various machine learning algorithms employed to evaluate their performance in predicting cardiovascular disease events using the Framingham dataset. “Results and comparison” Section presents the conclusions drawn from the research findings. “Conclusion” section discusses the future directions of the study, highlighting potential extensions, improvements, and practical implications for the pharmaceutical domain.

## Literature review

The burden of cardiovascular diseases (CVD) remains a major public health challenge worldwide, driving continuous research into risk prediction and prevention strategies. The Framingham Heart Study (FHS), a landmark longitudinal cohort study initiated in 1948, has laid the groundwork for understanding cardiovascular risk factors across generations^12^. While the original Framingham Risk Score continues to guide clinical decisions, recent advances in machine learning (ML) and deep learning (DL) have renewed interest in using the FHS dataset to build more accurate and individualized prediction models^13^.

Recent studies show that traditional regression models, although robust, may not fully capture the non-linear and high-dimensional interactions among risk factors in CVD^14^. Researchers like Alaa and Schaar^15^ and Alkhanbouli^16^ have demonstrated that ensemble ML models such as random forests and gradient boosting machines can outperform logistic regression when applied to cardiovascular datasets, including Framingham-derived data. These models leverage their ability to detect complex patterns, variable interactions, and hidden risk clusters, making them well suited for modern predictive health tasks.

The application of deep learning further expands these capabilities. Lundberg et al.^17^ highlighted that deep neural networks (DNNs) could surpass traditional risk scores for predicting all-cause mortality when combining clinical and imaging data. Ribeiro et al.^18^ extended this by using deep survival models to generate individualized risk trajectories for patients, showing that the integration of time-to-event modeling with DL adds value over static models. These findings indicate that using DL with FHS data could reveal new insights about how risk factors evolve over time, informing both clinical and policy interventions^19^.

However, the black-box nature of many ML and DL models raises significant concerns in healthcare, where transparency, trust, and accountability are critical^20^. This has spurred rapid growth in the field of explainable artificial intelligence (XAI). Tools such as SHAP (SHapley Additive ex Planations) and LIME (Local Interpretable Model-Agnostic Explanations) have become widely adopted for interpreting complex model predictions^21,22^. Caruana et al.^23^ and Samek et al.^24^ argue that XAI is no longer optional but necessary for responsible AI deployment in high-stakes areas like cardiovascular risk prediction.

In practical terms, combining ML, DL, and XAI helps balance predictive performance with interpretability^25^. Vimbi et al.^26^ showed how improved SHAP implementations make it feasible to apply explainability to large-scale clinical datasets such as Framingham. Researchers have also explored new explainability frameworks, including counterfactual explanations and causal feature attribution, to further enhance trust in automated decision support systems^27,28^. These developments highlight the growing emphasis on model transparency as a core requirement in predictive health analytics.

While these advances address clinical prediction challenges, the pharmaceutical sector increasingly recognizes the value of health data for optimizing commercial decision-making. Marketing mix modelling (MMM) remains a cornerstone for evaluating and forecasting the impact of key marketing variables such as promotion, pricing, placement, and product positioning^29,30^. In the pharmaceutical industry, MMM traditionally relies on aggregate sales data and marketing spend to estimate ROI, but it often overlooks patient-level clinical insights that could enable more precise targeting.

Emerging studies point to the potential of integrating health risk predictions into MMM. Xia et al.^31^ showed that supplementing MMM frameworks with external predictive signals improves forecast accuracy for new product launches. More recently, Kumar et al.^32^ discussed how big health data and predictive analytics are reshaping pharma’s approach to segmentation and resource allocation. Thomas and Frew^33^ further emphasized the growing importance of real-world evidence in guiding value-based pricing and market access strategies, which could benefit from robust clinical risk predictions.

Bringing these strands together, Tonekaboni et al.^34^ and Doshi-Velez and Kim^35^ highlight how personalization and data-driven marketing are transforming the pharmaceutical sector. Companies increasingly seek to move beyond broad, mass-market campaigns toward more targeted engagement strategies that align with individual patient risk profiles and treatment pathways. The integration of ML-based risk scoring into MMM frameworks supports this transition by identifying high-risk patient clusters who may benefit most from specific treatments or interventions^36^.

However, the direct connection between clinically validated risk models, explainable AI, and MMM remains underexplored in current literature. Existing works often treat these components in isolation—predictive models for clinical insights, XAI for interpretability, and MMM for marketing effectiveness^37^. Few studies provide an end-to-end framework demonstrating how insights from a dataset like Framingham can flow through predictive models, be interpreted through XAI, and inform evidence-based marketing decisions that are both profitable and patient-centered^38,39^.

This integration is not just a technical challenge but also an ethical imperative^40^. Vellido^41^ and Topol^42^ emphasize that transparent, explainable pipelines are critical for maintaining trust, meeting regulatory requirements, and supporting informed consent when patient data is used for commercial applications. This aligns with broader trends toward value-based healthcare and patient-centered marketing, where evidence-based engagement must be justifiable and compliant with privacy standards such as GDPR and HIPAA^43^.

In summary, the literature reveals four robust pillars: the enduring relevance of the Framingham Heart Study for cardiovascular prediction, the proven gains in predictive power through ML and DL methods, the necessity of XAI for transparency, and the well-established role of MMM in pharmaceutical strategy. Yet, integrating these pillars into a single, coherent pipeline that connects patient risk insights to real-world marketing actions remains an underdeveloped area—a gap this study aims to address.

By bridging these domains, this research contributes to the emerging field of precision marketing in healthcare, demonstrating how robust, interpretable CVD risk predictions from the Framingham dataset can drive more effective, ethical, and patient-centred pharmaceutical marketing decisions. Such integration holds promise for improving treatment adoption where it is needed most, maximizing ROI, and ensuring that data-driven innovation aligns with clinical and ethical standards.

Further some specific studies and methodologies that are relevant to the Framingham Heart Study (FHS) dataset work are mentioned below (Table 1):

**Table 1:** Comparative summary of relevant studies and techniques.

Study/source	Dataset used	Methodologies applied	Key outcomes	Relevance to your work
Dubey et al. (2024)	FHS	Stacked generalization with 7 ML classifiers; SMOTE balancing; IQR outlier detection	Achieved 97.2% accuracy on CHD prediction	Demonstrates ensemble learning and data balancing for high-performance modelling
EDA & ML Modelling (GitHub)	FHS	Exploratory Data Analysis; ML pipelines; visualization	Feature distribution insights; baseline ML models	Useful for initial data understanding and feature engineering
D’Agostino et al.	FHS	Framingham Risk Functions; multivariate regression	Developed 10-year CVD risk scores	Foundation for clinical risk modelling and therapeutic targeting
Framingham Risk Score Tools	FHS	Regression-based calculators for CHD, stroke, diabetes	Widely used in clinical practice	Can be adapted for marketing segmentation and outreach strategies
NHLBI & NIH Reports	FHS	Longitudinal cohort analysis; epidemiological modelling	Identified major CVD risk factors over decades	Validates the dataset’s richness and longitudinal value for predictive modelling

## Proposed methodologies

This research proposes a multi-phase methodological framework that integrates longitudinal epidemiological data from the Framingham Heart Study (FHS) with machine learning (ML), deep learning (DL), and explainable artificial intelligence (XAI) to generate actionable insights for pharmaceutical marketing. Various machine learning algorithms, including logistic regression, random forest, XG Boost and support vector machine are assessed for their predictive performance. In this research, advanced Deep Learning techniques, specifically Feedforward Neural Networks (FFNNs) and Multilayer Perceptron (MLPs), are applied to the Framingham Heart Study dataset to capture complex, non-linear relationships among cardiovascular risk factors. MLPs, which consist of multiple interconnected hidden layers and non-linear activation functions, are well-suited for structured clinical data and have been shown to outperform traditional linear models in predicting cardiovascular events. The predictive outputs of these models are explained using state-of-the-art Explainable AI (XAI) methods such as SHAP and LIME to ensure transparency and trustworthiness in individual risk predictions. The derived patient-level risk probabilities and their key contributing factors are then integrated into a Marketing Mix Modelling (MMM) framework. In this framework, the predicted risk segments (low, medium, high) are incorporated as moderators in hierarchical regression models alongside traditional marketing variables (e.g., promotional spending, channel allocation). This integration enables scenario-based simulations that help pharmaceutical companies optimize resource allocation, personalize outreach, and align promotional strategies with evidence-based patient needs. By combining robust Deep Learning predictions with XAI and MMM, this study aims to demonstrate a novel, data-driven approach for making more precise, ethical, and effective pharmaceutical marketing decisions. The primary dataset for this study is the Framingham Heart Study (FHS) cohort data, which contains longitudinal information on cardiovascular risk factors collected across multiple generations. Key variables will include demographic factors (age, sex), clinical measurements (blood pressure, cholesterol levels, BMI), lifestyle indicators (smoking status, physical activity), and outcome variables such as incidence of cardiovascular events and time-to-event information. To employ XAI (Explainable Artificial Intelligence) and ML (Machine Learning) Techniques for predicting mortality risk as well as cardiovascular disease (CVD) risk, the methodologies and materials used are as follows:

### Machine learning framework

**Fig. 1:**
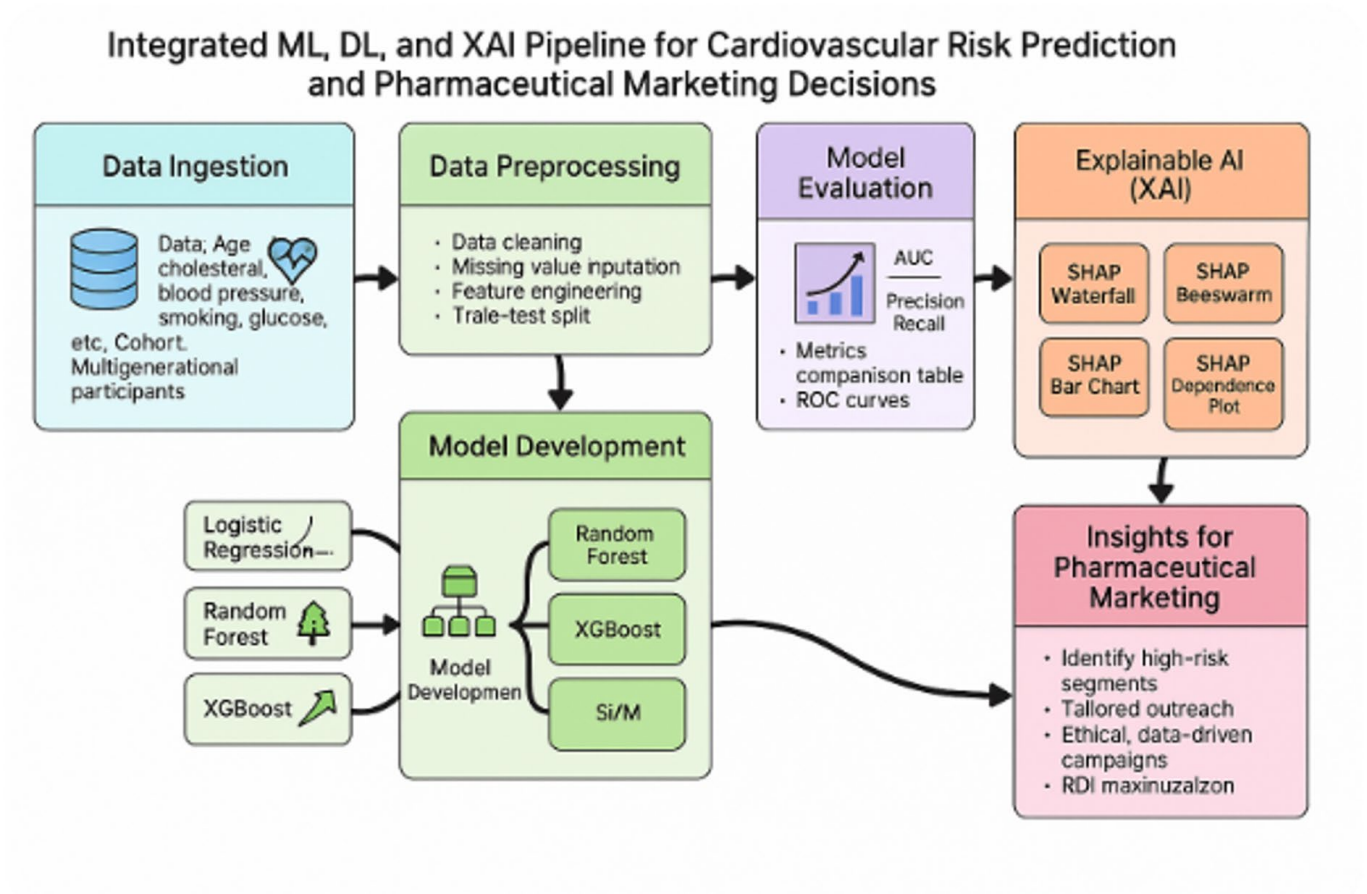
A hybrid ML, DL, and XAI approach using the Framingham Heart Study for precision pharmaceutical marketing.

In this research, we employ multiple supervised machine learning (ML) algorithms (Fig. 1) to predict cardiovascular risk using the Framingham Heart Study (FHS) dataset, demonstrating how robust predictive modelling can inform data-driven pharmaceutical marketing decisions. The selected models—Logistic regression, random forest, XGBoost, and support vector machine (SVM)—represent a balance between classical statistical methods, powerful ensemble techniques, and robust non-linear classifiers.

1. Logistic regression

Logistic regression is a foundational statistical model traditionally used in medical research and forms the basis of the original Framingham Risk Score (FRS). It models the probability of a binary outcome (such as presence or absence of cardiovascular disease) as a function of independent predictors like age, cholesterol level, blood pressure, and smoking status. Its primary strength lies in its interpretability—the regression coefficients directly indicate the direction and magnitude of association for each feature. In a pharmaceutical marketing context, this transparency helps stakeholders clearly understand which risk factors are most influential, supporting ethical targeting and risk communication.

2. Random forest

Random forest is an ensemble learning method that constructs a large number of decision trees and combines their outputs to improve prediction accuracy and control overfitting. Its ability to model complex, non-linear interactions among features makes it well-suited for the Framingham dataset, which includes both categorical and continuous variables with potential correlations. In the marketing domain, Random forest can uncover hidden patterns within patient segments, helping marketers identify high-risk groups that may benefit from tailored interventions or educational campaigns.

3. XG Boost

XG Boost (Extreme Gradient Boosting) is an advanced gradient boosting framework that builds decision trees sequentially, with each new tree correcting errors made by the previous ones. It is widely recognized for its scalability, computational efficiency, and high predictive performance on structured data. By capturing subtle non-linearities and complex feature interactions, XG Boost often outperforms simpler models, making it an ideal choice when high prediction accuracy is critical. In pharmaceutical marketing, this can enable more precise segmentation and targeted allocation of resources to maximize return on investment (ROI) and improve patient outcomes.

4. Support vector machine (SVM)

Support vector machine (SVM) is a powerful classifier that finds the optimal hyperplane to separate classes in high-dimensional space. SVMs are especially effective when the relationship between predictors and outcome is non-linear, thanks to kernel functions that project data into higher dimensions. Although SVM is not an ensemble by default, it is highly robust for binary classification tasks and serves as an important benchmark model. When used alongside other models in a stacking ensemble, SVM can contribute complementary strengths by capturing complex decision boundaries. For data-driven pharmaceutical marketing, SVM can help identify borderline cases where traditional linear models may fail, enhancing targeting precision for risk-based outreach or prevention campaigns.

### Comparative performance and stacking

To leverage the unique strengths of each algorithm, this study also explores a stacking ensemble approach. Here, logistic regression, random forest, XG Boost, and SVM act as base learners, with their individual predictions combined by a meta-learner to produce final predictions. This technique helps minimize the weaknesses of any single model while boosting overall prediction accuracy and stability—a key consideration for deploying AI in sensitive domains like healthcare and pharmaceutical decision-making.

Model performance was evaluated using standard classification metrics, including Accuracy, Precision, Recall, F1 Score, and Area Under the ROC Curve (AUC). Ensemble models and XG Boost typically delivered superior AUC and recall, while random forest provided strong stability and robustness. Logistic regression offered the highest transparency, and SVM added value where non-linear separations were evident.

### Implications for data-driven pharmaceutical marketing

Combining these diverse ML algorithms with Explainable AI (XAI) methods such as SHAP (SHapley Additive ex Planations) bridges the gap between black-box predictions and actionable, human-understandable insights. Pharmaceutical marketers can use these insights to understand which factors most strongly influence cardiovascular risk both globally and at the individual level, ensuring interventions are transparent, ethically sound, and highly targeted. This integrated ML framework illustrates how advanced predictive analytics, rooted in the Framingham study’s rigorous methodology, can drive personalized, trustworthy, and cost-effective marketing strategies—maximizing both patient benefit and marketing ROI.

By aligning classic statistical modeling, ensemble learning, and advanced classifiers within a unified, explainable ML pipeline, this research provides a robust template for integrating epidemiological insights into modern, data-driven pharmaceutical marketing.

### Deep learning framework

In addition to machine learning algorithms, this research integrates Deep Learning (DL) techniques to enhance cardiovascular risk prediction based on the Framingham Heart Study (FHS) dataset. While models like logistic regression, random forest, and XG Boost excel in structured health data tasks, Deep learning models (Fig. 1) offer the potential to capture complex, non-linear relationships and higher-order feature interactions that may otherwise remain hidden.

1. Feedforward neural network (FNN)

A Feedforward Neural Network (FNN) is the most basic form of an artificial neural network, consisting of an input layer, one or more hidden layers, and an output layer. Information flows in a single direction—forward—from input to output, without any cycles or feedback loops. For this study, the FNN is designed to predict binary cardiovascular risk (presence or absence of heart disease) using features such as age, cholesterol, blood pressure, smoking behaviour, glucose level, and more. The key strength of an FNN lies in its ability to approximate complex, non-linear functions. By using activation functions (e.g., Re LU, sigmoid) within hidden layers, the network can learn intricate relationships between risk factors that linear models might miss. This additional predictive capacity complements traditional ML models and helps validate the consistency of findings.

2. Multilayer perceptron (MLP)

A Multilayer Perceptron (MLP) is a specific type of FNN that includes multiple hidden layers, allowing the model to learn more abstract representations of the input data. In this research, the MLP architecture includes an input layer matching the number of Framingham risk factors, two or more hidden layers with appropriate neurons and activation functions, and a final output neuron with a sigmoid activation for binary classification. The MLP is trained using backpropagation with an optimizer like Adam or SGD to minimize the binary cross-entropy loss function. Its depth and non-linearity make it well-suited for capturing subtle patterns in patient risk profiles, especially when interactions among features are complex or non-additive.

Comparative Role of Deep Learning: While DL models such as FNNs and MLPs may not always outperform well-tuned ensemble methods like random forest or XG Boost on smaller tabular datasets, they add important value in several ways:

- *Model benchmarking*: DL models provide a non-linear, flexible benchmark to test whether simpler models capture the main risk patterns adequately.
- *Cross-validation*: If MLP results align closely with traditional models, this strengthens confidence in the overall modeling framework.
- *Explainability integration*: When paired with Explainable AI (XAI) tools such as SHAP, the hidden layers’ output can be interpreted to reveal how specific input features influence prediction outcomes, addressing the typical “black-box” criticism of neural networks.
- *Scalability*: MLPs can be easily extended or transferred to larger, more complex datasets or combined with other DL architectures (e.g., autoencoders) for future studies.
- By integrating DL models with the FHS dataset, this research demonstrates how neural networks can be used to uncover deeper risk stratifications. These insights can support precise targeting, personalized preventive strategies, and evidence-based messaging in pharmaceutical marketing campaigns. For example, identifying complex, high-risk patient subgroups allow marketers to tailor interventions or education programs with maximum impact and efficiency.
- Furthermore, combining DL predictions with stacking ensemble techniques alongside traditional ML models ensures robustness and minimizes reliance on any single modelling paradigm.

***Methodologies: The steps involved are as follows (***Fig. 1***)***.

*Step 1: Data acquisition*

- Source: Framingham Heart Study dataset.
- Load raw cohort data with demographics, clinical variables, and CVD outcomes.

*Step 2: Data preprocessing*

- Handle missing values.
- Feature engineering (risk scores, derived variables).
- Normalization & encoding.
- Train-test-validation split.

*Step 3: Predictive modelling*

- *Baseline*: Logistic regression.
- *Machine learning*: Random forest, XGBoost, SVM etc.
- *Deep learning*: Feedforward DNN, multi-layer perceptron.

*Step 4: Model evaluation*

- Metrics: AUROC, F1-score, Accuracy, Precision etc.
- Calibration plots.
- Compare all models.

*Step 5: Explainable AI layer*

- Apply SHAP for global/local explanations.
- Use LIME for instance-level insights.
- Visualize with Partial Dependence Plots.

*Step 6: Risk segmentation*

- Define risk groups.
- Cluster patients by predicted risk.

*Step 7: Marketing mix modeling (MMM)*

- Include marketing spend, channel mix, external factors.
- Integrate risk segments.

*Step 8: Scenario & ROI analysis*.

- Evaluate patient benefit vs. marketing efficiency.

*Step 9: Ethical & regulatory compliance*

- Check transparency, and responsible marketing guidelines.

***Materials***:

- *Computational Resources*: Advanced computing resources are employed to handle the computational demands of model training and validation.
- *Software Tools*: The study utilizes leading ML libraries (scikit-learn, Tensor Flow, Py Torch, Py Survival) and XAI packages (SHAP, LIME) for model development and interpretation.
- *Datasets*: This research uses the Framingham dataset, which contains longitudinal health and risk factor data collected over several decades. The dataset provides a rich source of structured variables for training and validating machine learning and deep learning models to predict cardiovascular disease (CVD) events and related outcomes.
- *Visualization Tools*: This study employs Matplotlib and Seaborn for conducting exploratory data analysis (EDA) and for generating clear visualizations of model predictions, performance metrics, and XAI explanations to support interpretability and insight extraction.

**Fig. 2:**
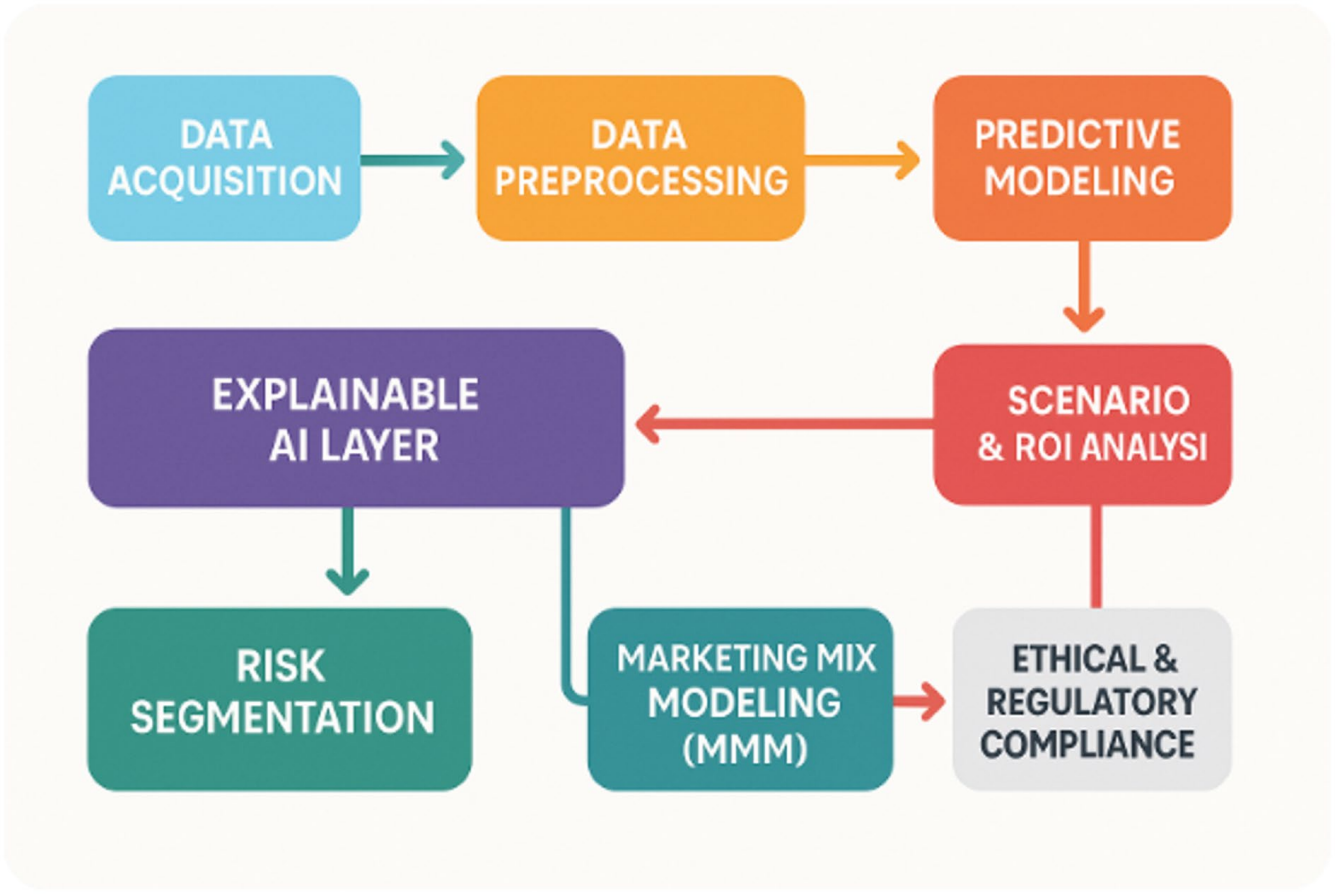
Comprehensive AI-driven analytics workflow integrating predictive modeling and XAI.

By integrating these methods (Fig. 2) and resources, researchers can effectively leverage machine learning, deep learning, and explainable AI techniques to enhance the reliability, transparency, and interpretability of predictive analytics within the context of Marketing Mix Modelling, using insights derived from the Framingham Heart Study for data-driven pharmaceutical marketing decisions.

### Fundamental aspects of the dual prediction process

The Framingham Heart Study (FHS) provides a rich, multi-generational longitudinal dataset capturing key demographic, lifestyle, and clinical variables, making it a cornerstone resource for developing and validating machine learning and deep learning models for cardiovascular disease prediction and related pharmaceutical marketing strategies.

**Fig. 3:**
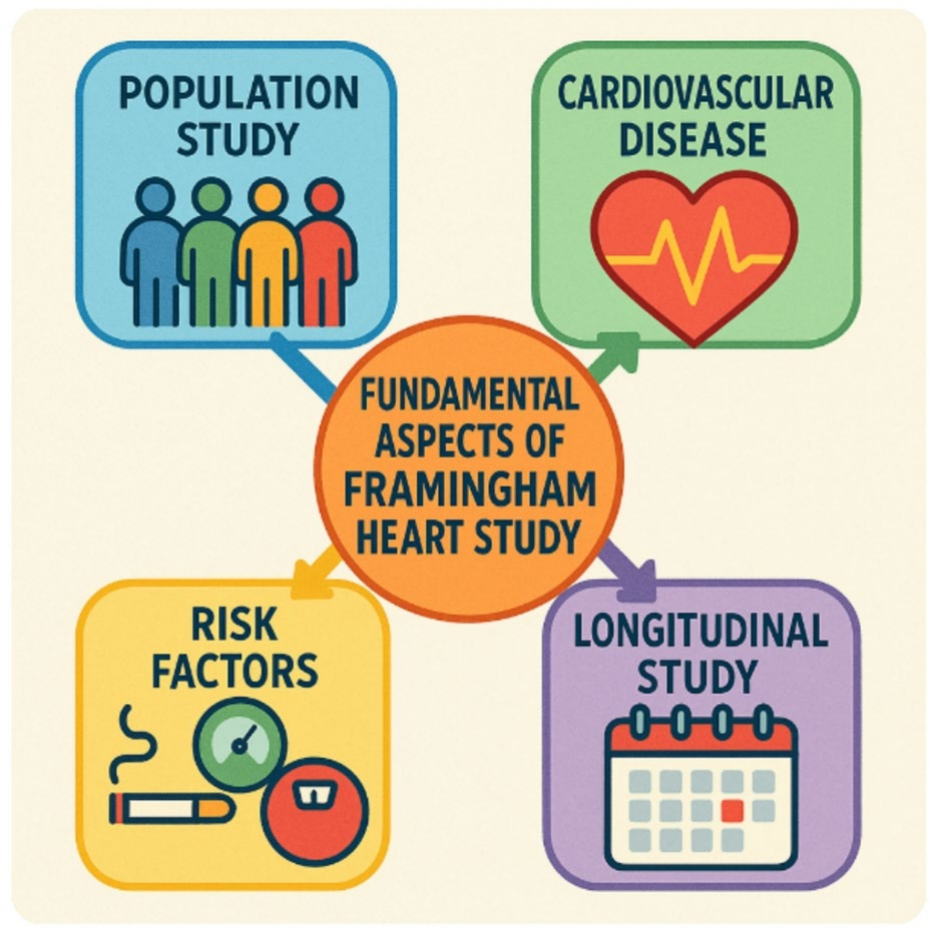
Core pillars and key insights of the Framingham Heart Study: a comprehensive look at cardiovascular health, risk factors, and longitudinal population research.

**Fig. 4:**
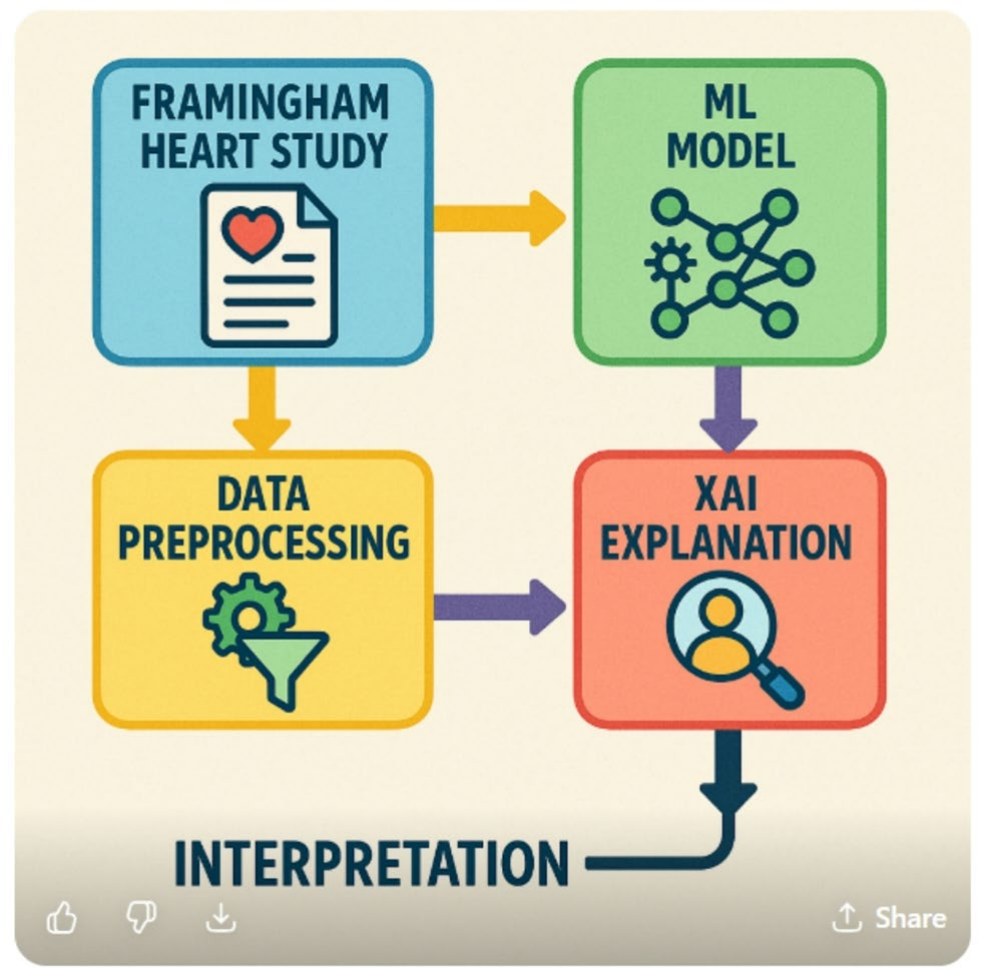
Visual process of applying XAI and ML techniques with the Framingham Heart Study.

The above diagram (Fig. 3) illustrates the fundamental pillars of the Framingham Heart Study, a landmark longitudinal research project on cardiovascular health. It highlights the study’s focus on population-based tracking, identification of key risk factors, and prevention of heart disease. The visual process (Fig. 4) further represents the dynamic flow of data and insights gathered over decades. This comprehensive approach continues to shape global guidelines for cardiovascular disease prevention and healthy living.

### Marketing mix modelling technique

**Fig. 5:**
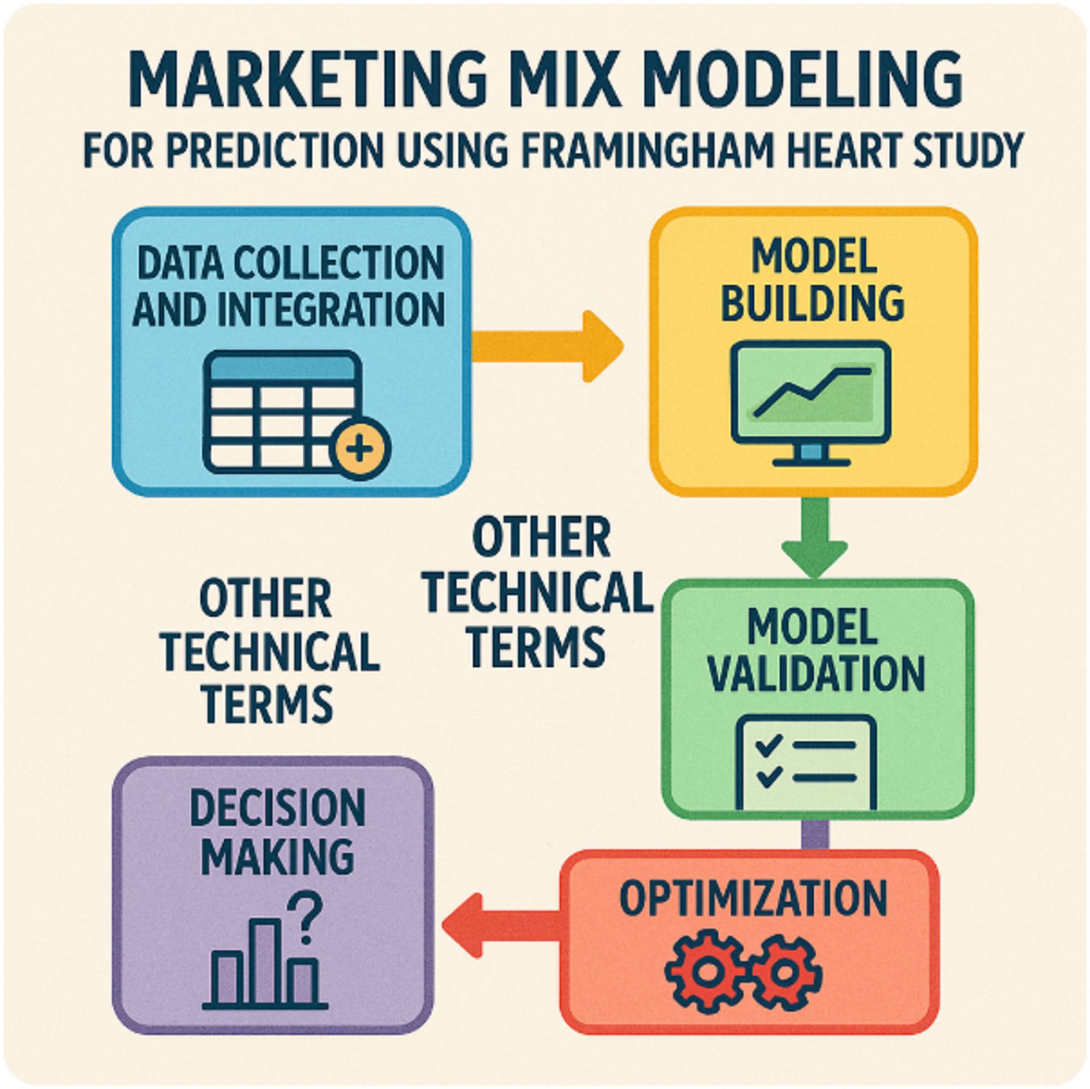
Applying marketing mix modeling to the Framingham Heart Study for predictive health insights.

Marketing Mix Modeling (MMM) is a quantitative analytical technique adapted here to evaluate how different strategies and interventions can influence health outcomes using insights from the Framingham Heart Study (Fig. 5). By analyzing decades of population health data, MMM helps researchers and policymakers understand the impact of various health promotion activities, risk factor management, and preventive measures on key health indicators^44,45^. This data-driven approach supports optimizing resource allocation, forecasting disease trends, and making informed public health decisions to improve cardiovascular outcomes and overall community well-being.

Here are the fundamental aspects of MMM:

1. Data collection & integration:
  Gather comprehensive longitudinal health data (e.g., demographics, lifestyle, medical history) from the Framingham Heart Study.
  Integrate additional data sources, such as interventions, awareness campaigns, or policy measures.
2. Variable identification & segmentation:
  Identify key predictors and risk factors (e.g., cholesterol levels, smoking, hypertension).
  Segment the population into relevant groups (age, gender, risk categories).
3. Model building:
  Develop statistical or ML-based models to quantify how different health interventions or risk factors influence cardiovascular outcomes.
  Use regression, time series, or ensemble methods for robust estimation.
4. Model validation & calibration:
  Test the model against historical trends to ensure accuracy and reliability.
  Refine the model using cross-validation and diagnostic checks.
5. Optimization & scenario analysis:
  Simulate different intervention strategies (e.g., smoking cessation programs, awareness campaigns).
  Optimize resource allocation to maximize health impact and cost-effectiveness.
6. Insights & decision making:
  Translate model outputs into actionable insights for policymakers, healthcare providers, and public health planners.
  Guide preventive strategies and policy decisions to improve population health outcomes.
7. Monitoring & continuous improvement:
  Continuously update the model with new Framingham data and emerging risk factors.
  Adapt strategies based on real-world results and changing population dynamics.

### Explainable AI

Explainable Artificial Intelligence (XAI) is a collection of methods and techniques aimed at making the predictions of machine learning models understandable and interpretable for people^46^. In the context of the Framingham Heart Study, XAI helps researchers, clinicians, and policymakers clearly see how complex AI models arrive at predictions about cardiovascular risk and health outcomes. By closing the gap between model complexity and the need for transparency, XAI builds trust, ensures accountability, and supports informed decision-making for better preventive strategies and patient care. Here are the fundamental aspects of XAI:

1. Data understanding & preparation
  Use the rich longitudinal Framingham dataset, which includes demographics, clinical measurements, lifestyle factors, and medical outcomes.
  Ensure data quality, handle missing values, and prepare features for interpretable modeling.

2. Transparent model selection
  Choose models that balance predictive power and interpretability—e.g., decision trees, rule-based models, or interpretable linear/logistic regressions.
  For more complex models (e.g., neural networks, ensemble models), plan to add explanation layers.

3. Feature importance analysis
  Use techniques like SHAP (SHapley Additive exPlanations) or LIME (Local Interpretable Model-agnostic Explanations) to quantify how much each risk factor contributes to predictions.
  Highlight the impact of key variables like cholesterol, smoking, age, and blood pressure.

4. Local & global explanations
  Provide global explanations to show overall model behaviour and risk factor relationships for the entire study population.
  Generate local explanations to clarify individual patient predictions and enable personalized insights.

5. Visualization & communication
  Use clear visual tools such as partial dependence plots, feature importance graphs, or force plots to communicate how predictions are made.
  Make outputs understandable to clinicians, policymakers, and patients.

6. Model validation & trust building
  Validate explanations with domain experts (cardiologists, epidemiologists) to ensure clinical relevance and trust.
  Compare explanations with established medical knowledge to detect unexpected patterns.

7. Ethical & regulatory compliance
  Ensure explanations support ethical decision-making and comply with regulations around transparency and fairness in health predictions.
  Use XAI to detect and mitigate biases in the model’s predictions.

8. Continuous feedback & improvement
  Incorporate expert feedback to refine explanations and models over time.
  Update explanation frameworks as new data and risk factors emerge from the ongoing Framingham study.

**Fig. 6:**
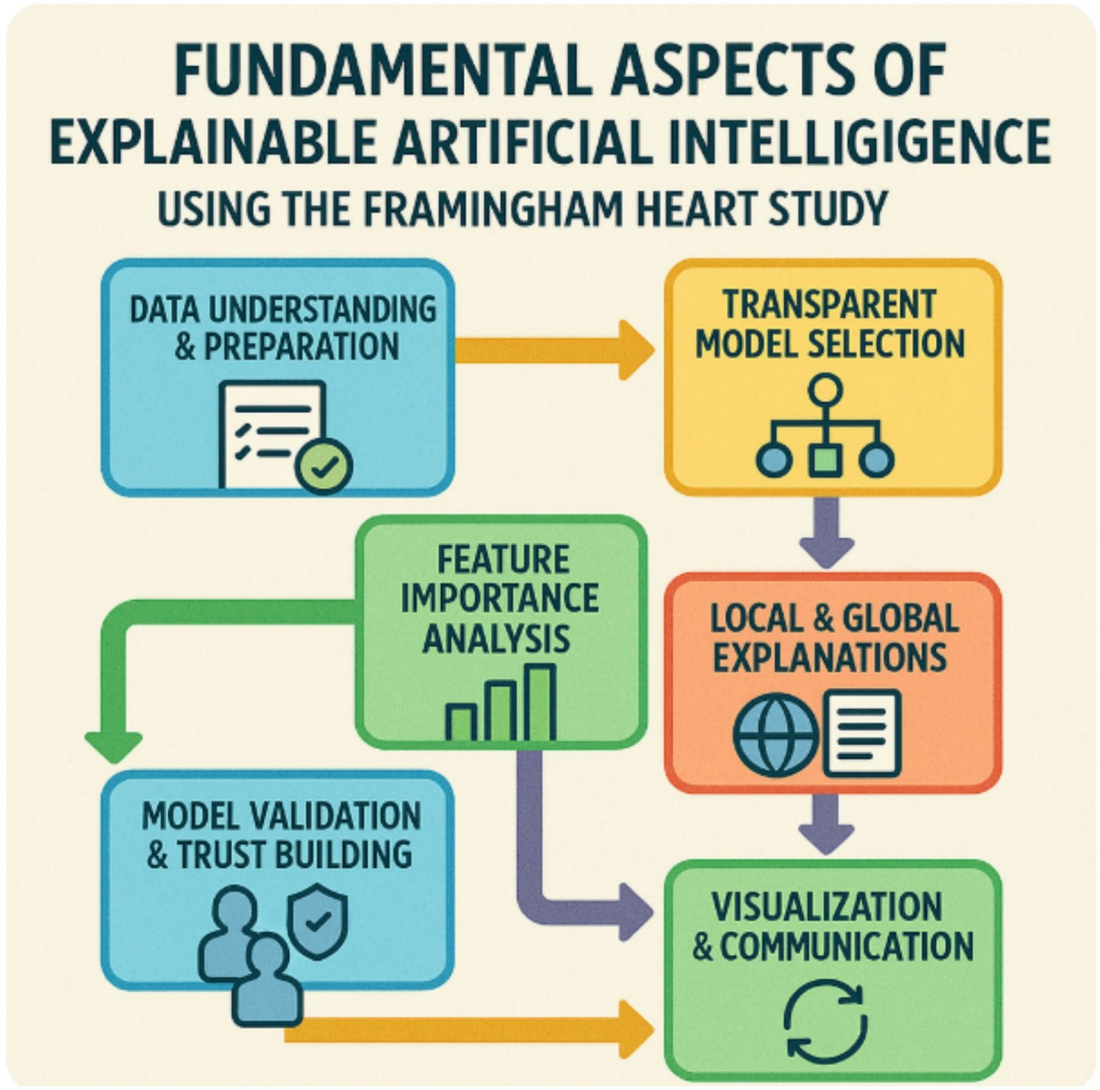
Explainable artificial intelligence for predictive modeling using the Framingham Heart Study.

The above diagram (Fig. 6) illustrates the essential steps of the XAI process applied to the Framingham Heart Study: collecting and preparing health data, training predictive models, generating risk predictions, adding an explainability layer, and interpreting the results to support clear, trustworthy insights into cardiovascular risk factors and outcomes.

***Explainable AI important techniques:***

#### SHAP (SHapley Additive exPlanations) algorithm

The following formula is used to determine the SHAP value for individual attribute x_i_ provided a model for prediction f and a particular instance x with characteristics x_1_, x_2_,…, x_n_:

i. *Marginal contribution*: For each feature xi, calculate its marginal contribution to the prediction f(x) by contrasting the forecast alongside and without the characteristic x_i_^47^.
ii. *Shapley value*: The mean of the marginal contributions of feature x_i_ across all potential feature subsets is its Shapley value.

*Mathematically*,* stepwise explanation*:

1. Select the model.
  Choose the trained machine learning model f(x) you want to explain.
2. Pick the instance.
  Choose the data point x(x) for which you want to explain the prediction.
3. Compute the baseline prediction.
  Calculate the expected value of the model across all data:

$${\phi _0}={\mathbb{E}}[f(x)]$$

4. Enumerate all feature subsets.
  For each feature i, consider all subsets S⊆N∖{i}, where N is the set of all features.
5. Compute the marginal contribution.
  For each subset S, compute:

$$f(S \cup \{ i\} ) - f(S)$$

This measures how much feature i adds to the prediction when added to subset S.

6. Assign a Shapley weight to each subset.

Use the formula:

$${w_S}=\frac{{\left| S \right|!(n - \left| S \right| - 1)!}}{{n!}}$$

7. Aggregate the weighted contributions.
  For each feature i, calculate:

$${\phi _i}=\sum\limits_{{S \subseteq N\backslash \{ i\} }} {{w_S} \cdot \left[ {F(S \cup \{ i\} ) - f(S)} \right]}$$

8. Repeat for all features.
  Compute SHAP values ϕ_i_ for every feature in the input x.
9. Sum the SHAP values to get the prediction.
  Combine all contributions:

$$f(x)={\phi _0}+\sum\limits_{{i=1}}^{n} {{\phi _i}}$$

#### LIME (Local interpretable model-agnostic explanations) algorithm

The LIME algorithm provides local justifications for each prediction produced by intricate machine learning models. Here’s a simplified explanation of the mathematical algorithm behind LIME-.

*LIME Algorithm*.

1. *Local perturbation*:
  Generate a new dataset by perturbing the instance xx for which we want an explanation. This involves creating new samples around x by randomly perturbing its features within a defined neighbourhood.

2. *Model training on perturbed data*:
  Use the involved sample to train an interpretable model. This simpler model is used to approximate the complex model’s local behaviour surrounding the instance x.

3. *Determining the significance of the attributes*:
  Determine each feature’s significance in the comprehensible model. This is accomplished by calculating the relative contribution of each feature to the interpretable model’s prediction.

4. *Formation of descriptions*:
  Creating an explanation for the complex model’s prediction for instance x using the feature importance values. The description identifies the characteristics that had the biggest impact on the outcome.

*Mathematically*,* stepwise explanation*:

1. Select model and instance
  Let f: R^n^→R: be a black-box model.
  Choose an instance x∈R^n^ whose prediction f(x) we want to explain.

2. Generate perturbed samples
  Create a set of samples {z_1_, z_2_, …, z_m_} around x, by randomly perturbing x’s features.
  Each z_i_ ∈ R^n^ is a variation of x.
  Use a binary interpretable version of inputs: x′∈{0,1}^n^, where 0 indicates the feature is “masked”.

3. Get model predictions
  For each perturbed sample z_i_, compute f(z_i_), the prediction from the black-box model.

4. Compute proximity measure
  Define a proximity kernel π_x_(z), which assigns a higher weight to points closer to x:

$${\pi _x}(z)=\exp \left( { - \frac{{D{{(x,z)}^2}}}{{{\sigma ^2}}}} \right)$$

where D is a distance metric (e.g., cosine or Euclidean), and σ is a width parameter.

5. Train a local interpretable model
  Define a simple, interpretable model g∈G (like linear regression), and fit it locally by minimizing:
    $$\mathcal{L}(f,g,{\pi _x}=\sum\limits_{{{z_i} \in Z}} {{\pi _x}({z_i}) \cdot {{(f({z_i}) - g(z_{i}^{\prime}))}^2}+\Omega (g)}$$
  zi′​ is the interpretable binary version of z_i_​.
  Ω(g): regularization term to ensure sparsity or simplicity (e.g., L1 norm).
  The goal is to find:

$$\arg \mathop {\hbox{min} }\limits_{{g \in G}} \mathcal{L}(f,g,{\pi _x})$$

6. Interpret coefficients
  The fitted model g provides weights w_J​_ for each interpretable feature j, representing its local importance:
$$g(z^{\prime})={w_0}+\sum\limits_{{j=1}}^{n} {{w_j} \cdot z_{j}^{\prime }}$$
  These weights form the explanation for f(x) in the neighborhood of x.

7. Present top features
  Display the top k features with the largest absolute weights ∣w_J_∣, typically as a bar chart or table to highlight their effect (positive or negative).

### Mathematical representation: applied machine learning algorithms

#### Logistic regression

Logistic regression is a widely used statistical method for modelling the probability of a binary outcome based on one or more predictor variables^48^. In the context of the Framingham Heart Study, logistic regression is often used to predict the likelihood of developing cardiovascular disease (yes/no) based on risk factors like age, cholesterol levels, blood pressure, smoking status, and other health indicators.

This technique estimates the relationship between the input variables and the probability of the outcome, producing coefficients that can be interpreted to understand how each factor contributes to the risk^49^. Its simplicity, interpretability, and effectiveness make logistic regression a fundamental tool for risk prediction and explainable AI in healthcare.

*Mathematical representation for simple logistic regression*:

1. Model equation

*Linear combination*:

$$\:z={\beta}_{0}+{\beta}_{1}{x}_{1}+{\beta}_{2}{x}_{2}+\dots+{\beta}_{p}{x}_{p}$$

- $$\:{\beta}_{0}$$ = intercept.
- $${\beta}_{1},{\beta}_{2},\dots,{\beta}_{p}$$​ = coefficients.
- $${x}_{1},{x}_{2},\dots,{x}_{p}$$​​ = input features.

2. Sigmoid (Logistic) Function

To constrain output to [0,1]:

$$\widehat{y}=P\left(Y=1|X\right)=\sigma \left(z\right)=\frac{1}{1+ {e}^{-z}}$$

3. Hypothesis

The predicted probability that Y = 1:

$${h}_{\beta}\left(X\right)=\frac{1}{1+ {e}^{-({\beta}_{0}+{\beta}_{1}{x}_{1}+{\beta}_{2}{x}_{2}+\dots\:+{\beta}_{p}{x}_{p})}}$$

4. Cost function (Log-Loss)

We use **maximum likelihood estimation (MLE)**.

The cost for one sample:

$$\:J\left(\beta\:\right)=-[y\mathrm{log}\left(\widehat{y}\right)+\left(1-y\right)\:\mathrm{l}\mathrm{o}\mathrm{g}(1-\widehat{y}\left)\right]$$

Total cost for all samples:

$$\:J\left(\beta\:\right)=-\frac{1}{m}\sum_{i=1}^{m}\left[{y}^{\left(i\right)}\mathrm{log}\left({\widehat{y}}^{\left(i\right)}\right)+\left(1-{y}^{\left(i\right)}\right)\:\mathrm{l}\mathrm{o}\mathrm{g}\left(1-{\widehat{y}}^{\left(i\right)}\right)\right]$$

where:

m = number of samples.

5. Goal: Minimize the cost function

We find the best $\:\beta\:$ that **minimizes** $\:J\left(\beta\:\right)$.

6. Gradient Descent (Optimization)

Update rule for each parameter $\:{\beta}_{j}$ ​:

$$\beta _{j} {\text{:= }}\beta _{j} - \alpha \frac{{\partial J\left( \beta \right)}}{{\partial \beta _{j} }}$$

where:

- $$\:\alpha\:$$ = learning rate.

*Partial derivative for each parameter*:

$$\:\frac{\partial\:J\left(\beta\:\right)}{\partial\:{\beta}_{j}}=\frac{1}{m}\sum_{i=1}^{m}\left[\left({\widehat{y}}^{\left(i\right)}-{y}^{\left(i\right)}\right){x}_{j}^{\left(i\right)}\right]$$

7. Decision boundary

Classify output:

$$\:If\:\widehat{y}\ge\:0.5\Rightarrow\:Y=1,\:\:\:else\:Y=0$$

8. Final model

After convergence:

- Use learned $\:\beta\:$ to predict:
  - $$\:\widehat{y}=P(Y=1|X)$$
  - Classify using threshold.

#### Random forest

Random forest is an ensemble machine learning method that builds multiple decision trees and combines their outputs to make more accurate and robust predictions^50^. In the context of the Framingham Heart Study, a random forest can be used to predict the risk of developing cardiovascular disease by analyzing numerous health indicators like age, cholesterol, blood pressure, smoking habits, and other lifestyle factors.

Each tree in the forest is trained on a random subset of the data and features, which reduces overfitting and improves generalizability^51^. The final prediction is made by aggregating the results (e.g., majority voting for classification). Random forests are highly valued for their strong predictive performance and built-in measures of feature importance, making them useful in explainable AI applications to highlight which risk factors have the greatest influence on health outcomes.

*Mathematical representation of random forest*:

*Step 1: Bootstrapping*.

For each tree $\:{T}_{b}$​:

- Draw a bootstrap sample $\:{D}_{b}$​ by randomly sampling n data points with replacement from D.

*Step 2: Grow a Decision Tree on* $\:{D}_{b}$.

At each node of the tree:

- Randomly select mmm features (where m < d).
- Among these m features, find the best split using a criterion:
  - Classification: Gini or Entropy.
  - Regression: MSE.

Split the node using the best (feature, threshold) pair.

Repeat until a stopping condition is met (e.g., max depth, min samples).

1. Prediction

Let $\:{\widehat{y}}_{b}\left(x\right)$ be the prediction from tree $\:{T}_{b}$​.

Classification: Majority Voting.

$$\:\widehat{y}\left(x\right)=mode\:\left({\widehat{y}}_{1}\left(x\right),{\widehat{y}}_{2}\left(x\right),\dots\:,{\widehat{y}}_{B}\left(x\right)\right)$$

Regression: Average.

$$\:\widehat{y}\left(x\right)=\frac{1}{B}\sum_{b=1}^{B}{\widehat{y}}_{b}\left(x\right)$$

2. Feature importance

Feature importance is computed as the average reduction in impurity contributed by that feature across all trees.

For feature j:

$$\:Importance\left(j\right)=\frac{1}{B}\sum_{b=1}^{B}\sum_{splits\:on\:j}\varDelta\:{I}_{b}$$

where $\:\varDelta\:{I}_{b}$​ is the impurity decrease at each split using feature j.

3. Out-of-bag (OOB) error estimate

Each tree is trained on a bootstrap sample, so ~ 1/3 of the data is not used to train that tree (out-of-bag).

For each data point $\:{x}_{I}$​, collect predictions from trees that did not train on it, and compute accuracy or MSE.

$$\:OOB\:Error=Error\:\left({\widehat{y}}_{oob}\left({x}_{i}\right),{y}_{i}\right)$$

#### XGBoost

XGBoost (Extreme Gradient Boosting) is a powerful and optimized gradient boosting algorithm used for classification and regression tasks^52^. It improves upon standard Gradient Boosting by incorporating regularization, efficient computation, and better handling of missing values^53^. The key advantage of XGBoost is that it handles missing values efficiently and provide fast training with parallel processing.

*Mathematical representation of XGBoost*:

1. Problem setup

Given training data:

$$\:D=\left\{\left({x}_{1},{y}_{1}\right),\left({x}_{2},{y}_{2}\right),\dots\:,\left({x}_{n},{y}_{n}\right)\right\}$$

Our goal is to learn a prediction function:

$$\:{\widehat{y}}_{i}=F\left({x}_{i}\right)=\sum_{m=1}^{M}{f}_{m}\left({x}_{i}\right),\:\:\:\:{f}_{m} \in\:\mathcal{F}$$

where $\:\mathcal{F}$ is the space of regression trees.

2. Objective function

The regularized objective function at step t is:

$$\:{\mathcal{L}}^{\left(t\right)}=\sum_{i=1}^{n}l\left({y}_{i},{{\widehat{y}}_{i}}^{\left(t\right)}\right)+\:\sum_{m=1}^{t}{\Omega}\left({f}_{m}\right)$$

where:

- l: differentiable loss (e.g., squared error, log loss).
- Penalizes tree complexity (number of leaves T) and weight magnitude $\:{w}_{j}$​:

$${\Omega}\left(f\right)= \Upsilon T + \frac{1}{2}\lambda\:\sum_{j=i}^{T}{w}_{j}^{2}$$

3. Additive model (Forward Stagewise Boosting)

We build the model additively:

$$\:{{\widehat{y}}_{i}}^{\left(t\right)}={{\widehat{y}}_{i}}^{\left(t-1\right)}+\:{f}_{t}\left({x}_{i}\right)\:$$

4. Second-order taylor approximation

Expand the loss function around the current prediction:

$${\mathcal{L}}^{\left(t\right)}\approx\:\:\sum_{i=1}^{n}\left[l\left({y}_{i},{\widehat{y}}_{i}^{\left(t-1\right)}\right)+\:{g}_{i}{f}_{t}\left({x}_{i}\right)+ \frac{{1}}{{2}} {h}_{i}{f}_{t}^{2}\left({x}_{i}\right)\right]+\:{\Omega}\left({f}_{t}\right)$$

where:

- $$\:{g}_{i}=\frac{\partial\:l\left({y}_{i},{\widehat{y}}_{i}\right)}{\partial\:{\widehat{y}}_{i}^{\left(t-1\right)}}\:\:$$ (1st derivative).
- $$\:{h}_{i}=\frac{{\partial}^{2}l\left({y}_{i},{\widehat{y}}_{i}\right)}{\partial\:{\left({\widehat{y}}_{i}^{\left(t-1\right)}\right)}^{2}}\:$$ (2nd derivative).

5. Structure of each tree

Each function $\:{f}_{t}$ corresponds to a regression tree with:

- T leaves.
- A mapping $$\:q\::\:{\mathbb{R}}^{d}\:\to\:\:\left\{1,\:2,\dots\:,T\right\}$$.
- Leaf weights $$\:{w}_{j}\in\:\mathbb{R}$$.
- So, $$\:{f}_{t}\left(x\right)={w}_{q\left(x\right)}$$

6. Optimize tree output

For a fixed tree structure (i.e., fixed $\:q\left(.\right)$), group instances into leaves:

Let $$\:I\_j=\left\{i\:\right|\:q(x\_i\:)=j\}$$.

Define:

- $$\:{G}_{j}=\sum_{i\in\:{I}_{j}}{g}_{i}$$
- ​$$\:{H}_{j}=\sum_{i\in\:{I}_{j}}{h}_{i}$$.

Then the optimal weight for each leaf j is:

$$\:{w}_{j}^{*}=-\frac{{G}_{j}}{{H}_{j}+\:\lambda}$$

and the corresponding minimized loss is:

$$\:{\mathcal{L}}_{leaf}=-\frac{1}{2}\sum_{j=1}^{T}\frac{{G}_{j}^{2}}{{H}_{j}+\:\lambda}+ \Upsilon T$$

7. Greedy tree growing (Split Finding)

For growing the tree:

- Try all possible splits.
- For each split that partitions a node into left (L) and right (R):

$$\:Gain=\frac{1}{2}\left[\frac{{G}_{L}^{2}}{{H}_{L}+\:\lambda}+\frac{{G}_{R}^{2}}{{H}_{R}+\:\lambda}-\frac{{\left({G}_{L}+{G}_{R}\right)}^{2}}{{H}_{L}+\:{H}_{R}+\lambda}\right]-\:\gamma\:$$

Choosing the split with the highest positive gain.

8. Regularization and controls

- $\:\lambda\:$: L2 regularization on leaf weights (prevents overfitting).
- $\:\gamma\:$: Penalty for adding a new leaf (controls tree depth).
- Subsample: Use a fraction of training data per round.
- Colsample_bytree: Random feature subset per tree.
- Learning Rate $\:\boldsymbol{\eta}$: Shrinks contribution of each tree.

9. Final prediction

After M boosting rounds:

$$\:{\widehat{y}}_{i}=\sum_{m=1}^{M}{f}_{m}\left({x}_{i}\right)$$

#### Support vector machine

Support vector machine (SVM) is a supervised machine learning algorithm used for classification and regression tasks. In the Framingham Heart Study, an SVM can help classify whether an individual is at high or low risk for developing cardiovascular disease based on factors like age, blood pressure, cholesterol levels, smoking status, and other health variables. An SVM works by finding the optimal hyperplane that best separates data points into different classes (e.g., disease vs. no disease) with the maximum possible margin^54^. SVMs are powerful when dealing with complex, high-dimensional data and can use different kernel functions to handle non-linear relationships. Although SVMs are less inherently interpretable than simpler models, they can still be combined with Explainable AI techniques (like SHAP or LIME) to understand which features most influence the model’s predictions, supporting trust and transparency in cardiovascular risk assessment.

*Mathematical representation of support vector machine*:

1. Linear decision boundary

For linearly separable data:

The decision boundary (hyperplane) is defined by:

$$\:{w}^{T}x+b=0$$

where:

- w = weight vector (normal to hyperplane).
- b = bias (offset).
- x = input vector.

2. Classification rule
  Predict class:

$$\:If\:{w}^{T}x+b>0,\:then\:y=+1$$

$$\:If\:{w}^{T}x+b<0,\:then\:y=-1$$

3. Margin
  The margin is the distance between the hyperplane and the closest data points (support vectors).
  Goal: Maximize the margin to achieve better generalization.

4. Distance of a point to the hyperplane

For a point $$\:{x}^{\left(i\right)}$$:

$$\:Distance=\frac{|{w}^{T}{x}^{\left(i\right)}+b|}{\left|\right|w\left|\right|}$$

5. Constraints for perfect separation

Each data point must be correctly classified:

$$\:{y}^{\left(i\right)}\left({w}^{T}{x}^{\left(i\right)}+b\right)\ge\:1,\:\:\forall\:i$$

where:

$$\:{y}^{\left(i\right)}\in\:\left\{+1,-1\right\}$$

6. Optimization problem

Maximize margin ⇨ Minimize ∥w∥ subject to the constraints.

Equivalent problem:

$$\:\genfrac{}{}{0pt}{}{min}{w,b}\:\:\:\frac{1}{2}{\left|\right|w\left|\right|}^{2}\:subject\:to\:{y}^{\left(i\right)}\left({w}^{T}{x}^{\left(i\right)}+b\right)\ge\:1.$$

7. Lagrangian formulation (Primal Form)

Use Lagrange multipliers ($\:{\alpha}_{i}$​) to handle constraints:

$$\:L\left(w,b,\alpha\:\right)=\frac{1}{2}{\left|\right|w\left|\right|}^{2}-\sum_{i=1}^{m}{\alpha}_{i}\left[{y}^{\left(i\right)}\left({w}^{T}{x}^{\left(i\right)}+b\right)-1\right]$$

with $$\:{\alpha}_{i}\ge\:0.$$.

8. Dual Form (Key to Kernel Trick)

Solve the dual problem for computational efficiency:

$$\:\genfrac{}{}{0pt}{}{min}{\alpha}\:\:\:\:W\left(\alpha\:\right)=\sum_{i=1}^{m}{\alpha}_{i}\:-\frac{1}{2}\sum_{i=1}^{m}\sum_{j=1}^{m}{\alpha}_{i}{\alpha}_{j}{y}^{\left(i\right)}{y}^{\left(j\right)}{\left({x}^{\left(i\right)}\right)}^{T}{x}^{\left(j\right)}$$

subject to:

$$\sum_{i=1}^{m}{\alpha}_{i}{y}^{\left(i\right)}=0,\:\:\:\:{\alpha}_{i}\ge\:0.$$

Once solved:

$$w=\sum_{i=1}^{m}{\alpha}_{i}{y}^{\left(i\right)}{x}^{\left(i\right)}$$

Only points with $\:{\alpha}_{i}>0$ are support vectors.

9. Kernel Trick (Non-linear SVM)

- For non-linearly separable data, map x to higher dimension using $\:\varphi\:\left(x\right)$ and replace inner product:

$$\:K\left({x}^{\left(i\right)},{x}^{\left(j\right)}\right)=\varphi\:{\left({x}^{\left(i\right)}\right)}^{T}\varphi\:\left({x}^{\left(j\right)}\right)$$

- Common kernels:
  - Linear: $$\:K\left(x,z\right)={x}^{T}z$$
  - Polynomial: $$\:K\left(x,z\right)={\left({x}^{T}z+c\right)}^{d}$$
  - RBF (Gaussian): $$\:K\left(x,z\right)=exp\left(-\gamma\:{\left|\right|x-z\left|\right|}^{2}\right)$$

10. Soft margin SVM

When data is not perfectly separable, introduce slack variables $\:{\xi}_{i}$:

$$\:\genfrac{}{}{0pt}{}{min}{w,b,\xi}\:\:\:\:\frac{1}{2}{\left|\right|w\left|\right|}^{2}+C\sum_{i=1}^{m}{\xi}_{i}\:\:\:\:subject\:to\:\:\:\:\:{y}^{\left(i\right)}\left({w}^{T}{x}^{\left(i\right)}+b\right)\ge\:1-{\xi}_{i},\:\:\:\:\:{\xi}_{i}\ge\:0.\:$$

- C controls trade-off between margin size and misclassification.

11. Final prediction.

Once w and b are found:

$$\:f\left(x\right)=sign({w}^{T}x+b)$$

Or for kernels:

$$\:f\left(x\right)=sign\left(\sum_{i=1}^{m}{\alpha}_{i}{y}^{\left(i\right)}K\left({x}^{\left(i\right)},x\right)+b\right)$$

#### Feed forward neural network (FFNN)

Feed forward neural network is a type of artificial neural network where information moves in only one direction—from input nodes, through hidden layers, to the output layer—without any cycles or loops^55^. In the Framingham Heart Study, an FFNN can be used to predict an individual’s risk of developing cardiovascular disease by learning complex, non-linear relationships among multiple health variables such as age, cholesterol levels, blood pressure, smoking habits, and lifestyle factors. The network consists of interconnected neurons organized in layers. Each neuron processes inputs with learned weights and biases, applies an activation function, and passes the output to the next layer^56^. By adjusting these weights during training, the FFNN learns patterns in the data that improve its predictive accuracy^57^. While FFNNs are powerful for capturing complex patterns, they are often considered “black box” models^58^. This makes Explainable AI techniques (like SHAP or layer-wise relevance propagation) essential to interpret which inputs most strongly influence the network’s predictions, ensuring transparency and trust when using neural networks for health risk prediction.

*Mathematical representation of feed forward neural network*:

1. **Architecture**
  Layers:
    Input layer (takes features).
    One or more hidden layers.
    Output layer (predicts output).
  Each layer $\:l$ has:
    Weights $$\:{W}^{\left[l\right]}$$.
    Biases $$\:{b}^{\left[l\right]}$$.
    Activation function.

2. **Notation**
  $$\:X\in\:{\mathbb{R}}^{{n}_{x}}$$​: input features.
  $$\:Y\in\:{\mathbb{R}}^{{n}_{y}}$$: true output.
  $\:{A}^{\left[l\right]}$: activations at layer $\:l$.
  $\:{Z}^{\left[l\right]}$: linear combination at layer $\:l$.

3. **Forward propagation**

- Linear Combination.

For layer $$\:l$$:

$$\:{Z}^{l}={W}^{\left[l\right]}{A}^{\left[l-1\right]}+{b}^{\left[l\right]}$$

- $\:{W}^{\left[l\right]}$: weight matrix of layer $\:l$.
- $\:{b}^{\left[l\right]}$: bias vector of layer $\:l$.
- $$\:{A}^{\left[0\right]}=X$$

- Activation.

Apply non-linearity:

$$\:{A}^{l}={g}^{\left[l\right]}\left({Z}^{\left[l\right]}\right)$$

where $\:{g}^{\left[l\right]}$ is the activation function (ReLU, Sigmoid, Tanh, etc.).

4. **Example: 2-Layer FFNN**

Suppose:

- 1 hidden layer (with ReLU).
- 1 output layer (with Sigmoid for binary classification).

Hidden layer:

$$\:{Z}^{1}={W}^{\left[1\right]}X+{b}^{\left[1\right]}{\:\:\:\:\:\:\:A}^{\left[1\right]}=ReLU\left({Z}^{\left[1\right]}\right)$$

Output layer:

$$\:{Z}^{2}={W}^{\left[2\right]}{A}^{\left[1\right]}+{b}^{\left[2\right]}\:\:\:\:\:\:\:\widehat{Y}={A}^{\left[2\right]}=\sigma\:\left({Z}^{\left[2\right]}\right)$$

5. **Cost function**

Example for binary classification: Cross-Entropy Loss.

$$\:J=-\frac{1}{m}\sum_{i=1}^{m}\left[{y}^{\left(i\right)}\mathrm{log}\left({\widehat{y}}^{\left(i\right)}\right)+\left(1-{y}^{\left(i\right)}\right)\:\mathrm{l}\mathrm{o}\mathrm{g}\left(1-{\widehat{y}}^{\left(i\right)}\right)\right]$$

where:

- m = number of samples.
- $$\:{\widehat{y}}^{\left(i\right)}\:$$= network output.

6. **Backward propagation**

Compute gradients to update weights:

Output layer error:

$$\:d{Z}^{\left[2\right]}={A}^{\left[2\right]}-Y$$

$$\:d{W}^{\left[2\right]}=\frac{1}{m}d{Z}^{\left[2\right]}{\left({A}^{\left[1\right]}\right)}^{T},\:\:\:\:\:\:\:d{b}^{\left[2\right]}=\frac{1}{m}\sum\:d{Z}^{\left[2\right]}$$

Hidden layer error:

$$\:d{A}^{\left[1\right]}={\left({W}^{\left[2\right]}\right)}^{T}d{Z}^{\left[2\right]}$$

$$\:d{Z}^{\left[1\right]}=d{A}^{\left[1\right]}\:\circ\:\:{g}^{{\prime}\left[1\right]}\left({Z}^{\left[1\right]}\right)\:\:\:\:\:\:\:\left(\circ\:means\:elementwise\:multiplication\right)$$

$$\:d{W}^{\left[1\right]}=\frac{1}{m}d{Z}^{\left[1\right]}{X}^{T},\:\:\:\:\:\:\:d{b}^{\left[1\right]}=\frac{1}{m}\sum\:d{Z}^{\left[1\right]}$$

7. **Update weights**

Use Gradient Descent:

$$\:{W}^{\left[l\right]}={W}^{\left[l\right]}-{\alpha\:dW}^{\left[l\right]}\:\:\:\:\:\:\:{b}^{\left[l\right]}={b}^{\left[l\right]}-{\alpha\:db}^{\left[l\right]}$$

where:

- $$\:\alpha\:$$ = learning rate.

8. **Repeat**

- Repeat Forward → Compute Cost → Backward → Update for many epochs until cost function converges.

9. **Final prediction**

At inference:

$$\:\widehat{Y}={A}^{\left[L\right]}=network\:output.$$

For binary classification:

$$\:If\:\widehat{y}>0.5,\:\:\:then\:Y=1;\:\:\:\:\:else\:Y=0.$$

### Conceptual foundations of a stacking model

Stacking, or Stacked Generalization, is an ensemble learning technique that combines multiple different machine learning models to improve predictive performance^59^. Instead of relying on a single algorithm, stacking leverages the strengths of diverse models to capture complex patterns and reduce errors.

In the context of the Framingham Heart Study, stacking can integrate predictions from various base models—such as logistic regression, random forest, support vector machine (SVM), and feed forward neural networks—each of which may capture different aspects of cardiovascular risk factors.

The key idea is that each base learner makes its own prediction, and then a meta-learner (often a simple model like logistic regression) takes these predictions as input to produce the final output. This second-level model learns how to best combine the strengths and correct the weaknesses of the base models.

By blending models that have different biases and prediction strategies, stacking typically delivers more accurate and robust predictions than any single model alone—which is especially valuable for complex health risk prediction tasks like those in the Framingham Heart Study.

### Data collection and description

The Framingham Heart Study is a long-term prospective study of the etiology of cardiovascular disease among a population of free-living subjects in the community of Framingham, Massachusetts. The Framingham Heart Study was a landmark study in epidemiology in that it was the first prospective study of cardiovascular disease and identified the concept of risk factors and their joint effects. The study began in 1948 and 5,209 subjects were initially enrolled in the study. Participants have been examined biennially since the inception of the study and all subjects are continuously followed through regular surveillance for cardiovascular outcomes. Clinic examination data has included cardiovascular disease risk factors and markers of disease such as blood pressure, blood chemistry, lung function, smoking history, health behaviors, ECG tracings, Echocardiography, and medication use. Through regular surveillance of area hospitals, participant contact, and death certificates, the Framingham Heart Study reviews and adjudicates events for the occurrence of Angina Pectoris, Myocardial Infarction, Heart Failure, and Cerebrovascular disease. The enclosed dataset is a subset of the data collected as part of the Framingham study and includes laboratory, clinic, questionnaire, and adjudicated event data on 4,434 participants. Participant clinic data was collected during three examination periods, approximately 6 years apart, from roughly 1956 to 1968. Each participant was followed for a total of 24 years for the outcome of the following events: Angina Pectoris, Myocardial Infarction, Atherothrombotic Infarction or Cerebral Hemorrhage (Stroke) or death. Over the decades, the study has gathered detailed demographic, lifestyle, medical, genetic, and clinical data through physical exams, laboratory tests, surveys, and follow-up interviews. Participants have been examined every few years, generating longitudinal data covering factors like blood pressure, cholesterol, smoking status, diabetes, family medical history, diet, and physical activity. It supports health guidelines and policy-making worldwide, shaping preventive strategies that save millions of lives. The primary objective of the Framingham Heart Study dataset is to identify, quantify, and track the major risk factors that contribute to cardiovascular disease (CVD) over time. By collecting and analyzing detailed health, lifestyle, and medical data from multiple generations, the dataset enables researchers to predict an individual’s risk of developing heart disease, understand how different factors interact, and guide evidence-based prevention and treatment strategies. This objective supports the development of predictive models, risk scores, and public health guidelines that have helped transform the prevention and management of heart disease worldwide.

The Framingham Heart Study is one of the most well-known and longest-running epidemiological studies in the world. Launched in 1948 in Framingham, Massachusetts, USA., it began by collecting comprehensive health data from a large community cohort to understand the causes and risk factors for cardiovascular disease (CVD). This study utilizes data from publicly available repository. Much of this data has been digitized and made available through publicly accessible online datasets for researchers worldwide, under strict ethical and privacy guidelines. The rich dataset serves as a foundation for developing and testing machine learning models—including predictive, interpretable, and explainable AI techniques—to better understand and manage cardiovascular risk. The dataset consists of 12,628 rows and 45 columns. It covers various heart study dataset, useful for prediction, risk scoring, or machine learning. Some of the factors are as follows:

randid, totchol age, sysbp, diabp cursmoke cigpday bmi diabetes bpmeds heartrte glucose educ prevchd prevap prevmi prevstrk prevhyp time period hdlc ldlc etc.

Describing some of the variables:

RANDID: Unique identification number for each participant. Values range from 2448 to 999,312.

TOTCHOL: Serum Total Cholesterol (mg/dL). Values range from 107 to 696.

AGE: Age at exam (years). Values range from 32 to 81.

SYSBP: Systolic Blood Pressure (mean of last two of three measurements) (mmHg). Values range from 83.5 to 295.

DIABP: Diastolic Blood Pressure (mean of last two of three measurements) (mmHg). Values range from 30 to 150.

CURSMOKE: Current cigarette smoking at exam. 0 = Not current smoker (*n* = 6598), 1 = Current smoker (*n* = 5029).

CIGPDAY: Number of cigarettes smoked each day. 0 = Not current smoker. Values range from 0 to 90 cigarettes per day.

BMI: Body Mass Index, weight in kilograms/height meters squared. Values range from 14.43 to 56.8.

DIABETES: Diabetic according to criteria of first exam treated or first exam with casual glucose of 200 mg/dL or more. 0 = Not a diabetic (*n* = 11097), 1 = Diabetic (*n* = 530).

BPMEDS: Use of Anti-hypertensive medication at exam. 0 = Not currently used (*n* = 10090), 1 = Current use (*n* = 944).

HEARTRTE: Heart rate (Ventricular rate) in beats/min. Values range from 37 to 220.

GLUCOSE: Casual serum glucose (mg/dL). Values range from 39 to 478.

PREVCHD: Prevalent Coronary Heart Disease defined as pre-existing Angina Pectoris, Myocardial Infarction (hospitalized, silent or unrecognized), or Coronary Insufficiency (unstable angina). 0 = Free of disease (*n* = 10785), 1 = Prevalent disease (*n* = 842).

PREVAP: Prevalent Angina Pectoris at exam. 0 = Free of disease (*n* = 11000), 1 = Prevalent disease (*n* = 627).

PREVMI: Prevalent Myocardial Infarction. 0 = Free of disease (*n* = 11253), 1 = Prevalent disease (*n* = 374).

PREVSTRK: Prevalent Stroke. 0 = Free of disease (*n* = 11475), 1 = Prevalent disease (*n* = 152).

PREVHYP: Prevalent Hypertensive. Subject was defined as hypertensive if treated or if second exam at which mean systolic was > = 140 mmHg or mean Diastolic > = 90 mmHg. 0 = Free of disease (*n* = 6283), 1 = Prevalent disease (*n* = 5344).

TIME: Number of days since baseline exam. Values range from 0 to 4854.

PERIOD: Examination Cycle. 1 = Period 1 (*n* = 4434), 2 = Period 2 (*n* = 3930), 3 = Period 3 (*n* = 3263).

HDLC: High Density Lipoprotein Cholesterol (mg/dL). Available for Period 3 only. Values range from 10 to 189.

LDLC: Low Density Lipoprotein Cholesterol (mg/dL). Available for Period 3 only. Values range from 20 to 565.

#### Significance of data collection—Framingham Heart Study

The comprehensive and systematic data collection in the Framingham Heart Study is what makes it a cornerstone of cardiovascular research worldwide. By gathering extensive, high-quality, longitudinal health data over decades, the study has enabled researchers to identify key risk factors—such as high blood pressure, high cholesterol, smoking, obesity, and diabetes—that contribute to heart disease and stroke.

This rich dataset allows scientists to track how lifestyle, genetic, and medical factors interact over time, leading to groundbreaking insights and risk prediction models that have shaped global clinical guidelines for preventing and managing cardiovascular disease.

Today, the availability of this data for advanced analytics and AI modelling provides a trusted foundation for developing interpretable, explainable, and accurate predictive tools. This not only supports individual patient risk assessment but also informs public health policies, awareness programs, and preventive healthcare strategies that save lives.

### Exploratory data analysis of Framingham Heart Study

**Fig. 7:**
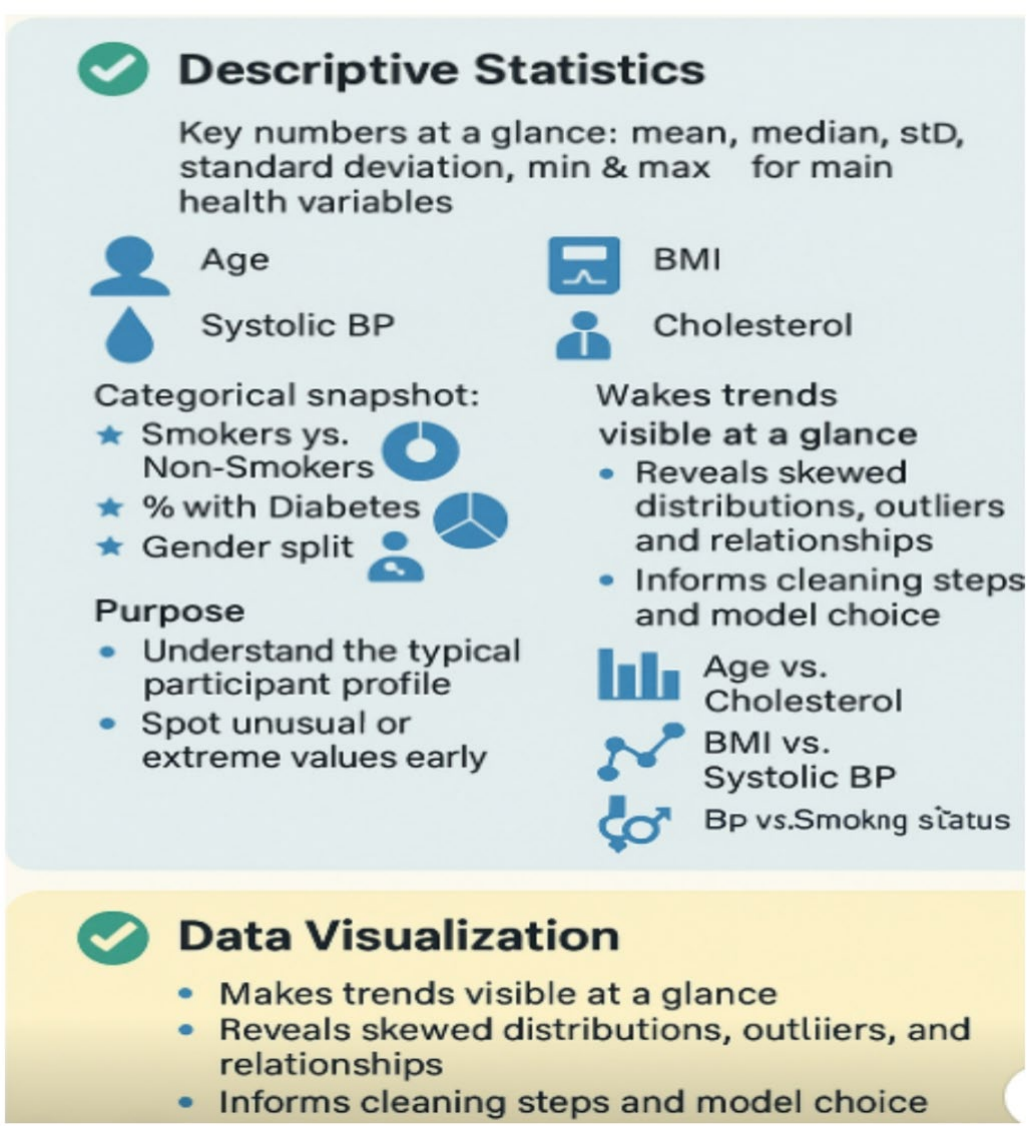
EDA snapshot of the Framingham Heart Study: descriptive statistics & visual insights.

Exploratory Data Analysis (EDA) is a crucial first step in working with the Framingham Heart Study dataset. It involves examining and summarizing the dataset’s main characteristics to uncover patterns, detect anomalies, test assumptions, and generate insights that guide further modelling (Fig. 7).

*Key EDA Steps with Framingham Data are as follows*:

- *Data inspection*:
  - Review variable types (numerical, categorical) such as age, cholesterol levels, smoking status, and blood pressure.
  - Check for missing values and outliers.
- *Descriptive statistics*:
  - Calculate measures like mean, median, standard deviation, and range for numerical features (e.g., systolic blood pressure, BMI).
  - Count frequencies for categorical features (e.g., smoker vs. non-smoker).
- *Data visualization*:
  - Use histograms and boxplots to show the distribution of continuous variables.
  - Create bar charts or pie charts for categorical features.
  - Apply scatterplots to explore relationships (e.g., cholesterol vs. age).
- *Correlation analysis*:
  - Generate a correlation matrix to identify relationships between risk factors (e.g., age and blood pressure).
  - Highlight strong positive or negative correlations relevant to heart disease.
- *Missing value treatment*:
  - Identify patterns of missing data and decide on imputation or removal.
  - Assess whether missingness is random or systematic.
- *Outlier detection*:
  - Detect extreme values that may affect model performance, using plots or statistical rules (e.g., z-scores, IQR).
- *Initial hypothesis formation*:
  - Use EDA findings to refine research questions or guide the choice of machine learning models.
- *Its matters*:
  - EDA of the Framingham dataset ensures that the data is clean, well-understood, and ready for accurate modelling.

and explainable AI applications—ultimately supporting trustworthy cardiovascular risk prediction.

### Methodological parallels between cardiovascular risk modeling and its possible applications in pharmaceutical

#### Marketing

The Framingham Heart Study (FHS), initiated in 1948, is a landmark longitudinal epidemiological study aimed at identifying the common factors contributing to cardiovascular disease. Over decades, it has evolved into a multigenerational cohort-based study, using statistical modeling to establish causal links between behavioral, biological, and environmental variables (e.g., cholesterol levels, blood pressure, smoking, diet, genetics) and cardiovascular outcomes. Its legacy lies not just in the discovery of specific risk factors but in the methodological framework it introduced—combining multivariate regression, risk scoring, and population-based inference to understand the predictive power and interplay of diverse features in a complex system.

#### Methodological parallels: risk scoring vs. ROI estimation

One of the most enduring contributions of the Framingham study is the Framingham Risk Score (FRS)—a multivariate scoring system that predicts a person’s 10-year risk of developing cardiovascular disease. It combines coefficients derived from statistical models with individual-level values of key predictors (e.g., age, cholesterol, blood pressure). In a marketing context, ROI models or contribution models serve an analogous function: they quantify how much each marketing input contributes to the final outcome (e.g., sales uplift or brand awareness), often resulting in dashboards or simulators for marketing planning. Both approaches require robust feature engineering and data preprocessing to make the model interpretable and reliable. FHS controls for population stratification, missing data, and cohort effects, while MMM accounts for media lags, adstock effects, and base sales. In both domains, analysts must deal with collinearity, endogeneity, and model overfitting—especially when historical variables are interdependent or influenced by unobserved factors. The use of regularization (e.g., Ridge or Lasso regression) in MMM today mirrors the variable selection and penalization techniques used in epidemiology. Furthermore, both models have evolved from simple regression to more complex machine learning techniques (e.g., decision trees, random forests, and ensemble models) while maintaining a commitment to transparency and actionable insights. Just as modern extensions of FHS use ML for risk prediction, MMM is now integrating Bayesian frameworks, time-series models, and XAI tools like SHAP or LIME to improve trust and usability among marketers.

Cross-Domain Insights: Causal Thinking and Decision Support - A critical aspect of the Framingham methodology is its focus on causality over correlation. While observational, the study designs incorporate longitudinal follow-up, temporality, and confounder control, which strengthens causal inference. Similarly, MMM must account for causal assumptions—for instance, determining whether an increase in TV ad spend caused the sales increase or merely coincided with other market forces. Researchers are increasingly integrating causal inference techniques (e.g., difference-in-differences, instrumental variables) to bridge the gap between correlation and causality in MMM.

Another shared objective is decision support. In public health, the FHS has influenced guidelines around statin therapy, smoking cessation, and blood pressure management. In marketing, MMM outputs guide budget allocation, channel optimization, and campaign timing. In both settings, models are used not just for retrospective analysis but for prospective “what-if” simulations—testing the impact of changes in key variables under different scenarios. This capacity for strategic forecasting makes both methodologies critical tools for policy and business stakeholders.

Incorporating the Framingham model’s structured, scientific mindset into marketing can enrich MMM’s interpretability and credibility. For instance, just as the FHS separates modifiable vs. non-modifiable risk factors, MMM can distinguish between controllable (marketing levers) and uncontrollable (weather, economy) drivers of performance. This helps marketers understand which variables they can act upon and which they must monitor.

While the current research does not include a full-scale Marketing Mix Modeling (MMM) implementation, its methodological insights offer a pathway for potential applications in pharmaceutical marketing analytics. The predictive and causal frameworks employed in cardiovascular risk modeling could, in future research, inform models assessing marketing effectiveness—such as evaluating the influence of promotional activities, channel engagement, or campaign sequencing. Once appropriate marketing expenditure and control variable data become available, similar analytical structures could be applied to measure the long-term impact and optimize strategic decision-making.

### Addressing potential data biases and domain specifics

- *Potential data biases:*
  - Demographic Limitations: The original Framingham cohort primarily consisted of white, middle-class residents of Framingham, Massachusetts. This limits generalizability to more diverse racial, ethnic, or socioeconomic groups.
  - Selection Bias: Participants who consented to long-term follow-ups may have better health behaviours than the general population, potentially underestimating risk in other groups.
  - Historical Context: Health behaviours and medical treatments have evolved since 1948; older data may not fully reflect modern risk factors or treatment effects.
- *Domain specific challenges:*
  - Longitudinal Nature: The dataset includes repeated measures over decades, which introduces challenges like missing follow-up data and changing measurement techniques.
  - Time-Varying Risk Factors: Some variables change over time (e.g., cholesterol, smoking status), requiring careful handling in predictive modelling.
  - Privacy & Ethics: Health data must comply with strict privacy rules; maintaining de-identification and ethical use is critical.
- *The way potential biases are addressed:*
  - Using additional cohorts to validate findings on more diverse populations.
  - Using imputation and robust statistical methods to handle missing or time-varying data.
  - Clearly communicating limitations when presenting risk predictions to avoid overgeneralization.
  - Applying sampling and weighting techniques to adjust for demographic imbalances.

## Results and discussions

Results for Dataset - The table and screenshots below represent the results obtained with respect to the Framingham Heart Study Dataset:

**Table 2:**
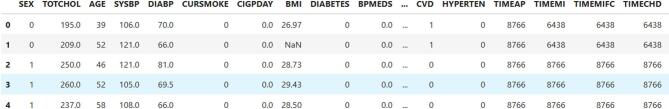
Comparative summary of relevant studies and techniques

Table 2 presents a snapshot of the cleaned and pre-processed dataset derived from the Framingham Heart Study, which serves as the foundation for building and evaluating the proposed Machine Learning (ML), Deep Learning (DL), and Explainable Artificial Intelligence (XAI) models. The table displays selected variables, including demographic attributes (e.g., SEX, AGE), clinical measurements (TOTCHOL for total cholesterol, SYSBP and DIABP for systolic and diastolic blood pressure, BMI for body mass index), lifestyle factors (CURSMOKE, CIGPDAY), and key health indicators (DIABETES, BPMEDS, CVD, HYPERTEN). Time-to-event variables (TIMEAP, TIMEMI, TIMECHD, etc.) are also included to support survival analysis and risk prediction tasks.

This dataset enables the integration of predictive modelling with interpretability techniques to identify at-risk populations and derive actionable insights for pharmaceutical marketing strategies, targeted interventions, and risk-based patient segmentation. Missing values, as observed in some variables (e.g., BMI), are addressed using appropriate data imputation techniques to ensure robust model performance and reliable explanations.

**Fig. 8:**
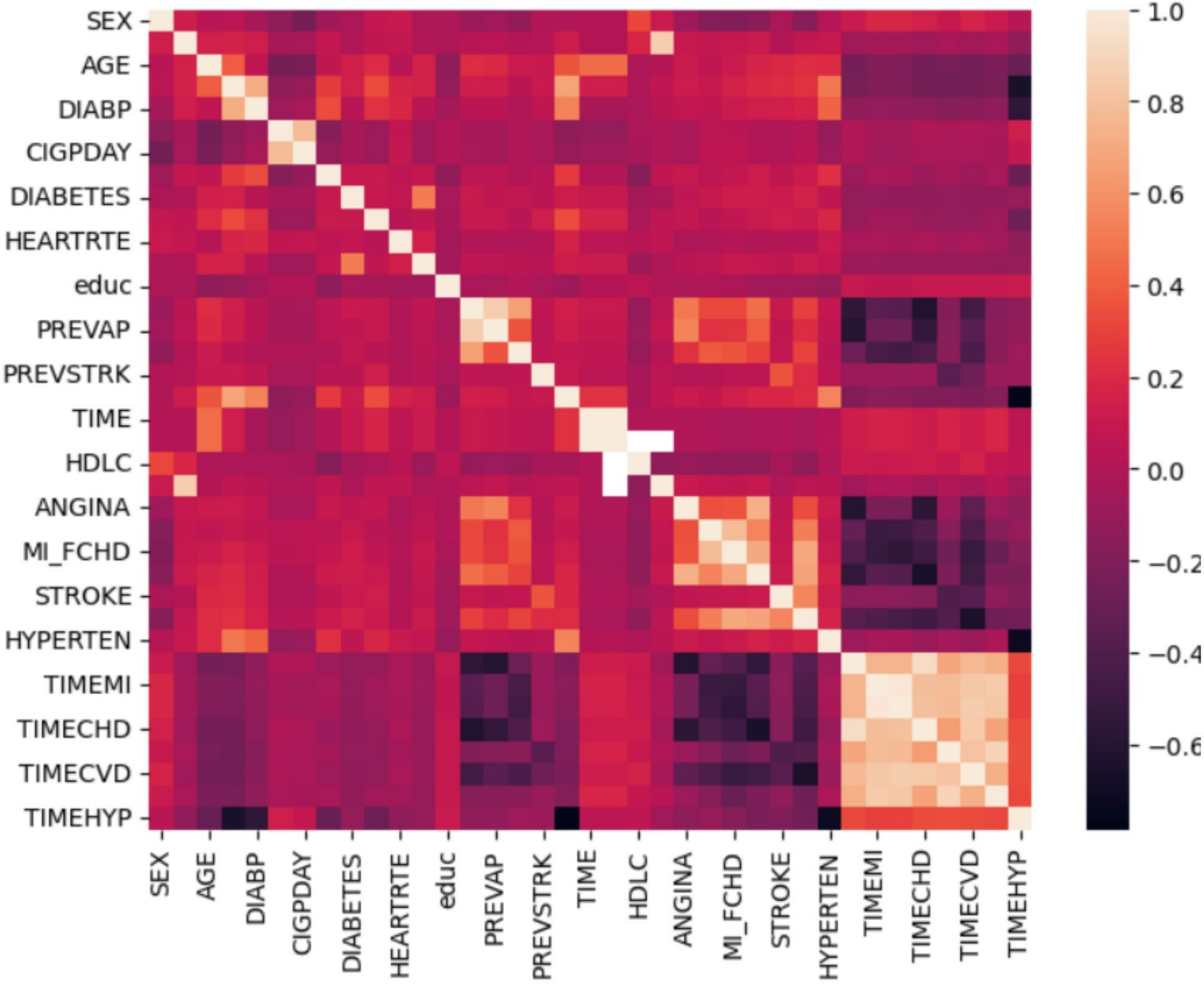
Correlation heatmap of key variables in the Framingham Heart Study dataset.

Figure 8 illustrates the correlation matrix of key variables derived from the Framingham Heart Study dataset, visualized as a heatmap. This plot highlights the pairwise linear relationships among demographic factors (e.g., SEX, AGE, educ), clinical and lifestyle variables (DIABP, CIGPDAY, DIABETES, HEARTRTE), cardiovascular risk indicators (ANGINA, MIFCHD, STROKE, HYPERTEN), and time-to-event outcomes (TIMEMI, TIMECHD, TIMECVD, TIMEHYP).

The color scale ranges from − 1 to + 1, where lighter shades indicate stronger positive correlations and darker shades represent stronger negative correlations. The matrix helps identify potential multicollinearity issues, influential predictors, and hidden patterns that can inform feature selection and model design.

This correlation analysis provides foundational insights for developing robust Machine Learning and Deep Learning models and enhances the explainability framework (XAI) by clarifying which features may most significantly influence prediction outcomes. Understanding these interdependencies supports more transparent, interpretable, and data-driven decision-making for targeted pharmaceutical marketing and risk-based customer segmentation. It shows there is high correlation among the time features as well as many missing values in HDLC / LDLC. There were also some variables that were related e.g. PREVAP and PRECHD.

**Fig. 9:**
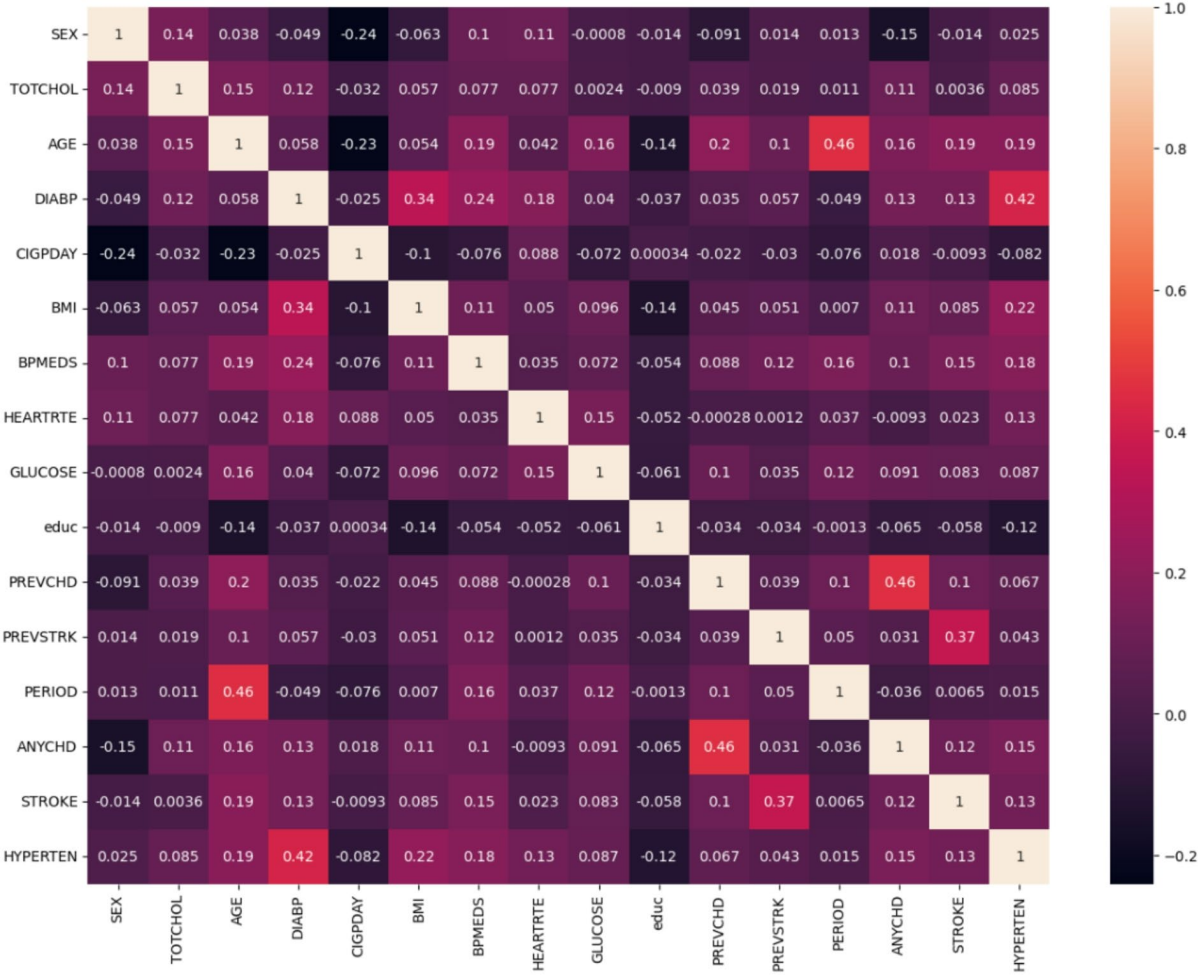
Refined correlation matrix of clinical and demographic variables in the Framingham Heart Study.

Figure 9 displays the refined comprehensive processed correlation matrix of selected demographic, clinical, and behavioural variables from the Framingham Heart Study dataset. This heatmap visualizes the pairwise Pearson correlation coefficients among key variables, including SEX, AGE, TOTCHOL (total cholesterol), DIABP (diastolic blood pressure), CIGPDAY (cigarettes per day), BMI, GLUCOSE, BPMEDS, HEARTRTE (heart rate), educ (education level), and various cardiovascular risk factors and historical conditions (PREVCHD, PREVSTRK, PERIOD, ANYCHD, STROKE, HYPERTEN).

The color gradient ranges from dark (negative correlation) to light (positive correlation), highlighting the strength and direction of linear relationships between variables. This visual exploration supports the identification of strongly correlated predictors, potential multicollinearity, and latent relationships within the data, which are critical for informed feature selection and model design. By mapping these interdependencies, this correlation analysis underpins the development of robust Machine Learning and Deep Learning models and strengthens the integration of Explainable AI (XAI) methods. Understanding these relationships also informs the segmentation of at-risk populations, enabling more precise targeting and actionable insights for data-driven pharmaceutical marketing strategies.

Any real concerning correlations is not seen and thus there is low risk of data redundancy. Visualization looks at key parameters of current data. The correlated variables are already eliminated to reduce the potential of data leakage and increase the possibility of determining feature importance. It helps in gaining a better intuition of the features left and how they interact with each other and the target variable. There are also slight correlations between some of the remaining features and it would be useful to infer why these features are showing relationships.

**Fig. 10:**
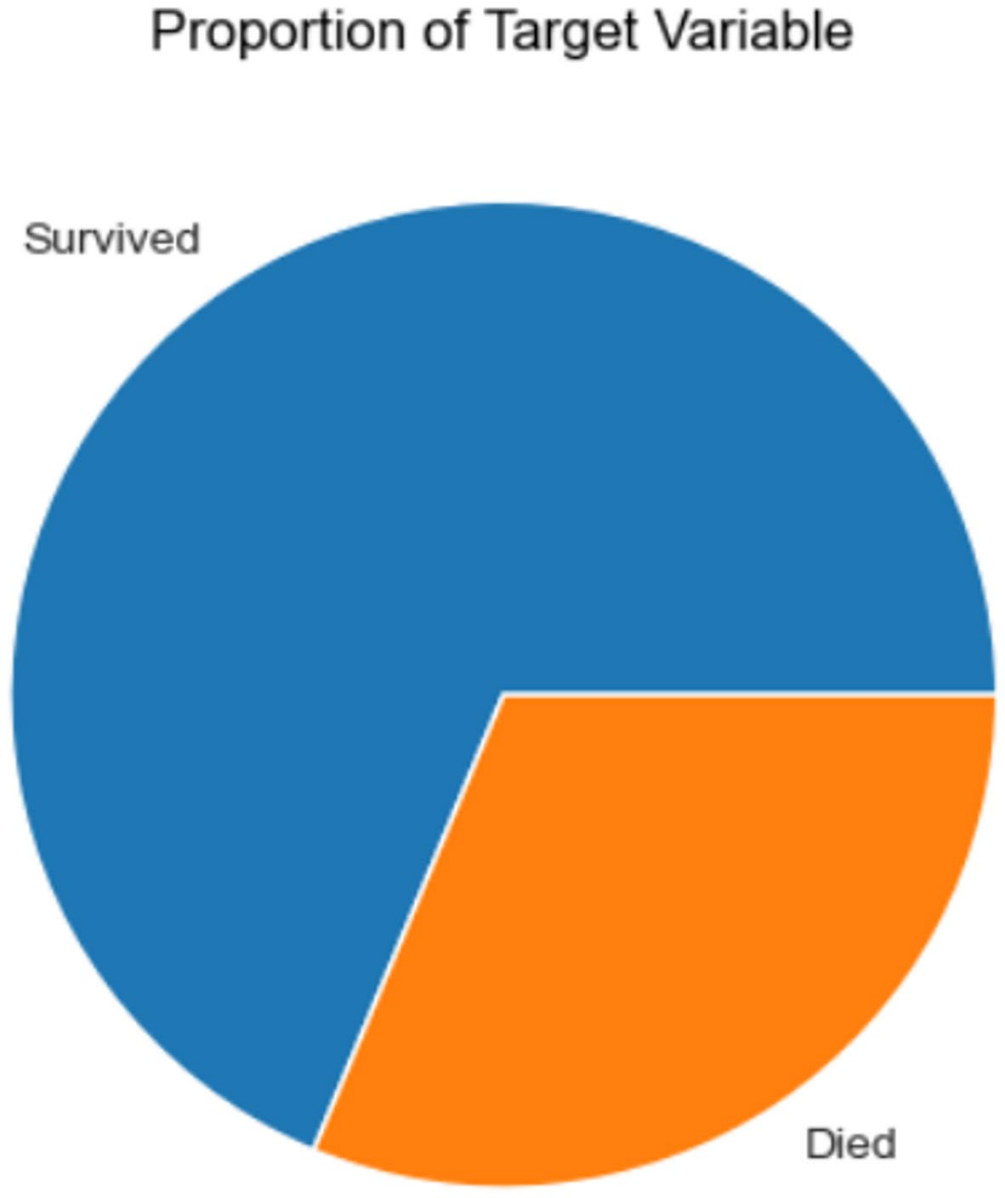
Proportion of survival and mortality outcomes.

Figure 10 shows the proportion of the target outcome variable in the Framingham Heart Study dataset, visualized as a pie chart. The chart illustrates the relative distribution between participants who survived and those who died over the study period. The larger blue segment represents the proportion of individuals who survived, while the smaller orange segment indicates those who experienced the event of interest (death) during the follow-up period. This class distribution provides essential context for developing and evaluating the Machine Learning (ML) and Deep Learning (DL) models used in this study. Understanding the imbalance in the target variable is crucial for selecting appropriate modelling strategies, addressing class imbalance during training, and ensuring the reliability and fairness of predictive outcomes. Cleary, majority of the patients survived. This shows that the classes are clearly imbalanced. Furthermore, by integrating Explainable AI (XAI) methods, the research aims to provide transparent insights into the factors influencing survival outcomes, supporting targeted pharmaceutical interventions and informed marketing decisions.

**Fig. 11:**
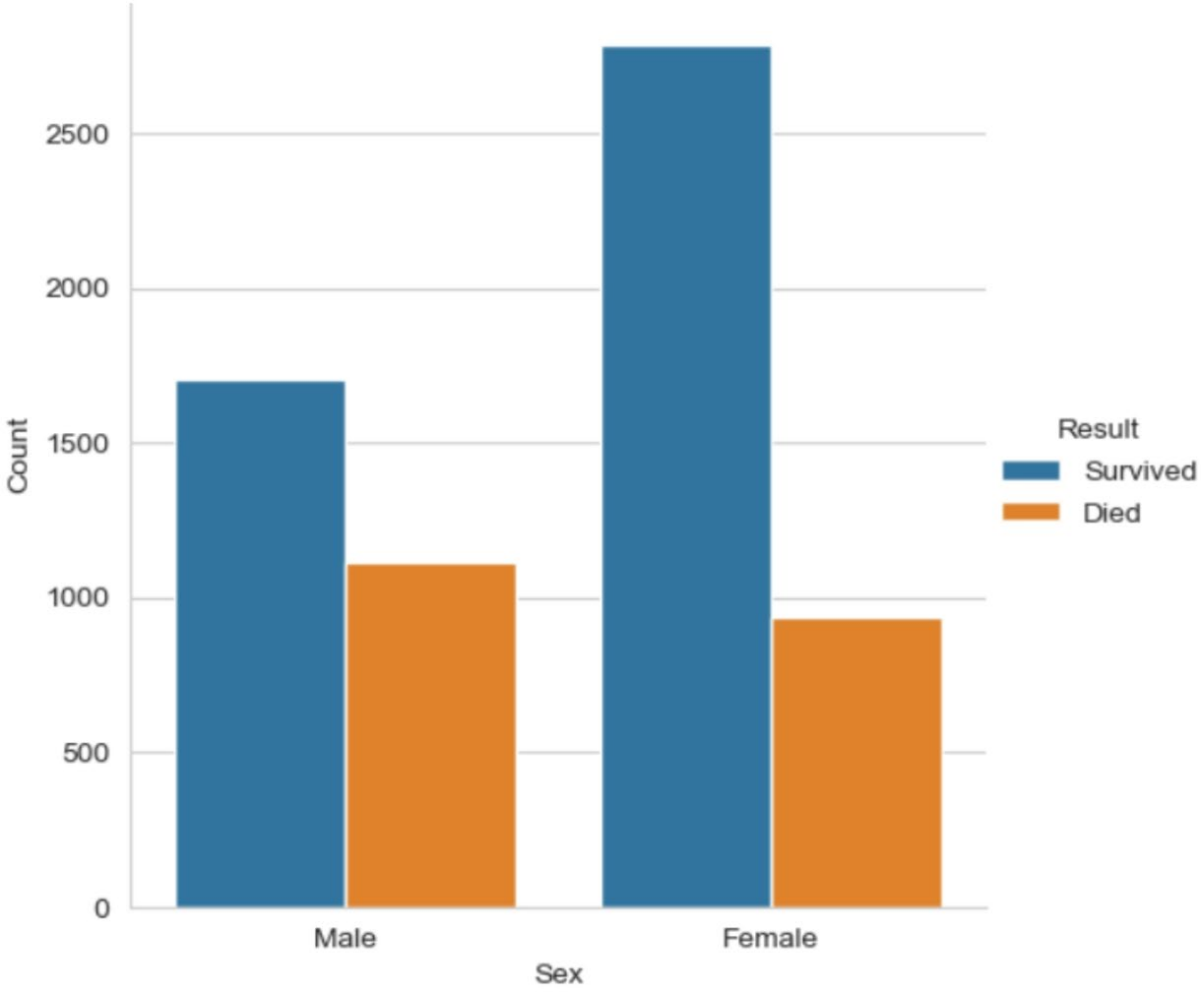
Distribution of survival and mortality by gender.

Figure 11 illustrates the distribution of survival outcomes by sex within the Framingham Heart Study dataset. The bar chart shows the count of male and female participants who survived (blue bars) and those who died (orange bars) during the study period.

This sex-based breakdown highlights demographic variations in survival status and provides useful context for building predictive Machine Learning (ML) and Deep Learning (DL) models. Understanding these demographic differences is crucial for designing fair and unbiased models and for applying Explainable AI (XAI) methods to interpret how sex may influence predicted health outcomes. These insights support more precise customer segmentation, risk stratification, and targeted pharmaceutical marketing strategies driven by transparent, data-informed decision-making.

**Fig. 12:**
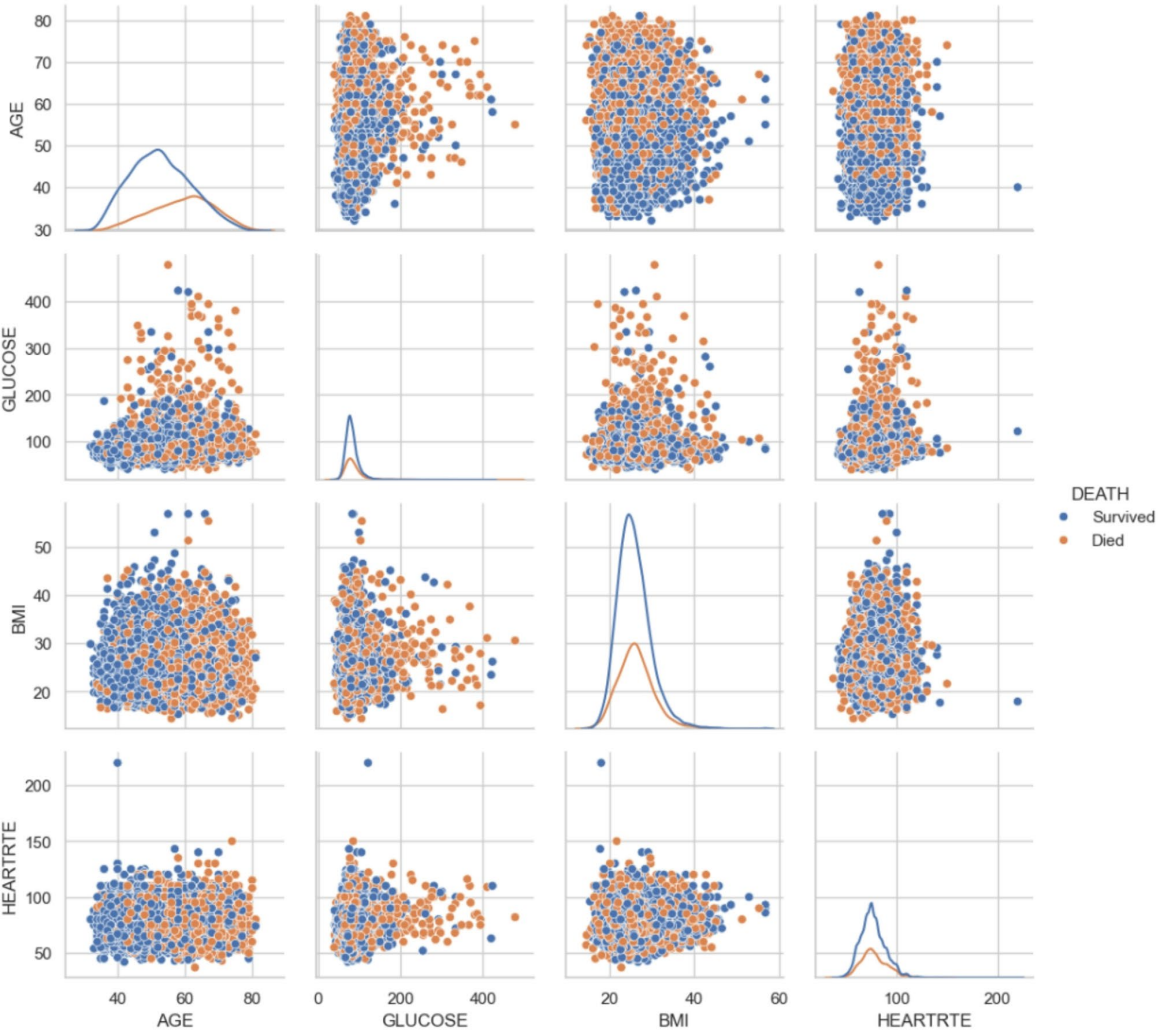
Scatter plot matrix of predictors with survival outcome labels.

Figure 12 presents a pair plot visualizing the relationships and distributions of key clinical variables—AGE, GLUCOSE, BMI, and HEARTRTE (heart rate)—in the Framingham Heart Study dataset, grouped by survival outcome. Each scatter plot shows the bivariate relationship between pairs of variables, with data points coloured by whether the participant survived (blue) or died (orange) during the study period. The diagonal plots display the univariate distribution of each variable for both outcome groups. There does not appear to be very strong trends between any of the variables. From what is shown, age and heart rate seem to correlate with a higher death count. We can also see that the distribution of people that died is skewed towards older people, implying that age increased the chance of death. The distributions of BMI and glucose show that whether someone lived or died had very little influence from these features. Another notable change is that as age, glucose and BMI increase, there is a slight trend for heart rate to increase. However, it is worth mentioning that the data is very dispersed and there isn’t a strong correlation.

This visual exploration highlights potential patterns, trends, and separability between individuals who survived and those who did not. For example, differences in the distribution curves suggest that age, glucose levels, BMI, and heart rate may vary systematically between the two groups.

These insights help guide feature selection and the design of robust Machine Learning (ML) and Deep Learning (DL) models. Furthermore, they lay the groundwork for integrating Explainable AI (XAI) techniques to interpret how these variables contribute to survival predictions. Understanding these interactions is essential for developing transparent risk stratification models and informing precision-targeted pharmaceutical marketing strategies based on data-driven insights.

**Fig. 13:**
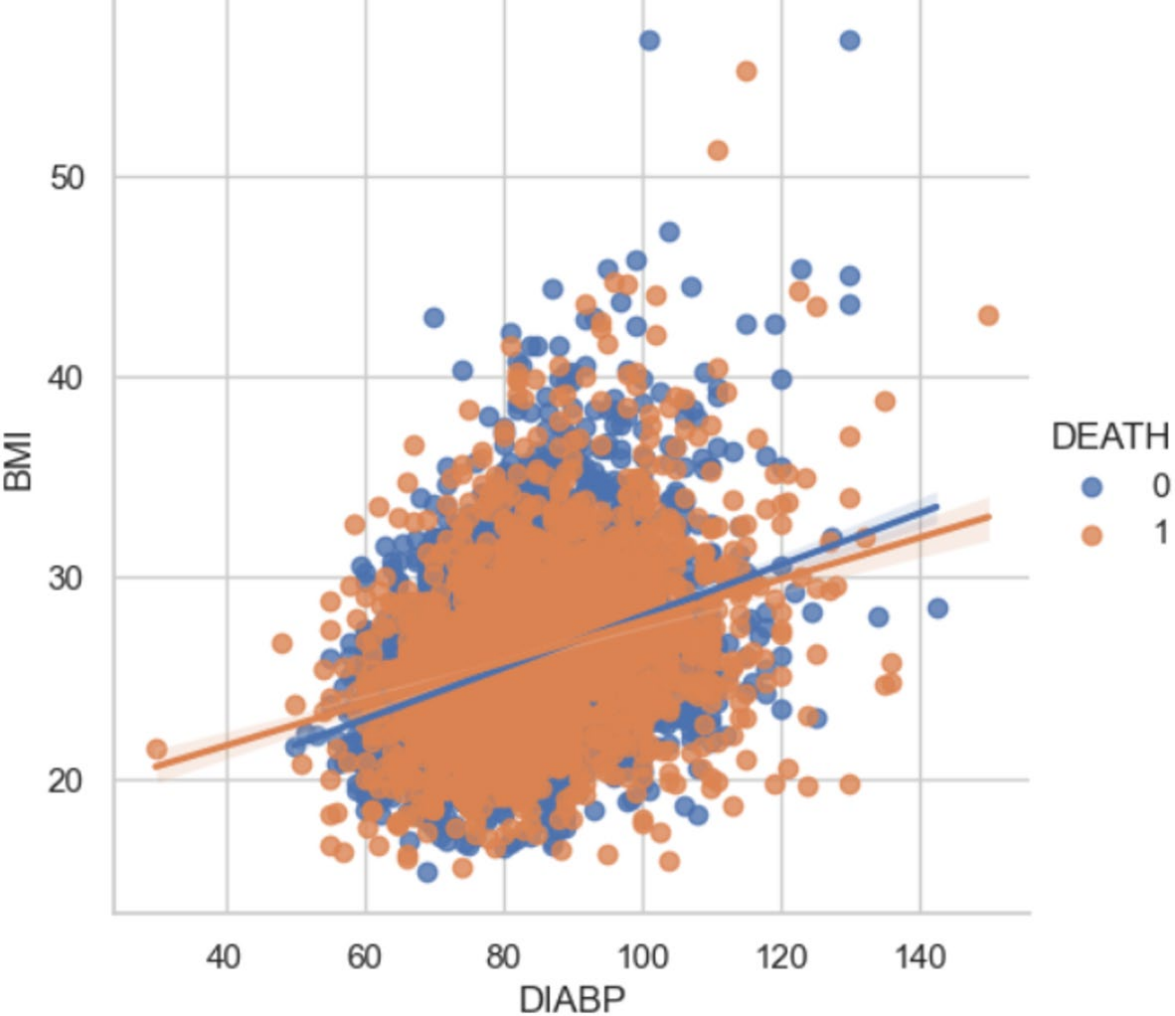
Scatter plot of DIABP (diastolic blood pressure) vs. BMI (body mass index (BMI) with outcome-based trend lines.

Figure 13 presents a scatter plot illustrating the relationship between Diastolic Blood Pressure (DIABP) and Body Mass Index (BMI) among participants in the Framingham Heart Study. Each point represents an individual participant, color-coded by their survival outcome: blue indicates participants who survived (DEATH = 0) and orange indicates those who died (DEATH = 1) during the study period.

The plot includes separate regression trend lines for each outcome group, providing a visual indication of the positive association between DIABP and BMI across survival statuses. This suggests that higher diastolic blood pressure may be correlated with higher BMI in both groups, although the strength and slope of this association vary slightly by survival outcome.

There seems to be a slight positive trend between Diastolic Blood Pressure and Body Mass Index. The trend is steeper with people who survived versus those who died. We can attribute this to causation: blood pressure increases with a higher Body mass Index. Bayesian Hyperparameter Optimisation is the chosen method. It has a few advantages that are worth mentioning. It will not brute force its way through the problem as other methods would. This means that we can still get quality results, efficiently.

In other words, this bivariate relationship highlights the potential combined influence of blood pressure and body composition on cardiovascular risk and mortality. Understanding this interaction is vital for developing predictive Machine Learning (ML) and Deep Learning (DL) models that accurately capture health risks. By applying Explainable AI (XAI) techniques, the contribution of variables like DIABP and BMI to predicted survival outcomes can be made transparent, supporting data-driven pharmaceutical marketing strategies that target relevant risk segments with greater precision.

**Table 3:** Summary of the optimal hyperparameters identified for the three ML algorithms applied.

S.No.	ML algorithm	Best loss	Time required
1.	XG boost	0.21146788990825693	It takes 2.6438944816589354 min
2.	Random forest	0.213914373088685	It takes 1.8111060897509257 min
3.	Logistic regression	0.21085626911314992	It takes 0.03584413131078084 min

The reported result (Table 3)—an XGB Classifier achieving a best loss of 0.2115—indicates that the optimized Extreme Gradient Boosting (XG Boost) model has a low classification error (or low loss) when predicting survival outcomes based on the Framingham Heart Study data. A loss of ~ 0.21 means the model is well-tuned and capable of learning meaningful patterns from the demographic and clinical features (like BMI, DIABP, AGE, etc.) to distinguish between participants who survived and those who did not during the study period. The relatively short tuning time (≈ 2.64 min) demonstrates that the chosen optimization approach is computationally efficient, which is practical when deploying or iterating models in real-world applications.

The random forest classifier achieved a best loss of approximately 0.214, indicating that after hyperparameter tuning, the model has a low classification error when predicting survival outcomes based on the Framingham Heart Study data. The short tuning time (~ 1.81 min) demonstrates that the random forest is efficient to optimize and is practical for iterative model development when working with large clinical datasets. A low loss value means the random forest is effectively capturing complex, non-linear patterns among the input features (AGE, BMI, DIABP, etc.) that help distinguish between patients who survived and those who did not.

The logistic regression model achieved a best loss of ~ 0.211, which indicates that, after tuning, its classification error is slightly lower than that of the random forest classifier and comparable to the XGB Classifier.

The extremely short tuning time (~ 0.036 min, or about 2 s) highlights the computational simplicity and speed of logistic regression—a major advantage for baseline modelling, iterative testing, and comparison with more complex ML and DL models. Despite its simplicity, Logistic regression can provide strong baseline performance and, combined with Explainable AI (XAI) methods, offers high interpretability, making it a valuable benchmark for explaining survival outcomes in the Framingham Heart Study.

Interpreting the table results as follows: Table 3 presents a comparative summary of the optimal hyperparameter tuning results for the three machine learning algorithms applied—XGB classifier, random forest classifier, and logistic regression—using the Framingham Heart Study dataset. Among the models tested, logistic regression achieved the lowest loss value (0.2109), indicating strong baseline predictive performance with minimal classification error. Remarkably, it required the least computational time (0.036 min), which highlights its efficiency and suitability as a robust, interpretable benchmark model. The XGB classifier and random forest classifier yielded slightly higher loss values (0.2115 and 0.2139, respectively), demonstrating competitive accuracy while capturing more complex, non-linear interactions among the risk factors. These models required moderately longer optimization times (2.64 min for XG Boost and 1.81 min for random forest), reflecting the additional computational complexity inherent in ensemble methods. These results demonstrate that while all three models perform well for predicting survival outcomes, the trade-off between model complexity, interpretability, and computational efficiency should be carefully considered when integrating Explainable AI (XAI) techniques and developing data-driven strategies for pharmaceutical marketing. Specifically, the logistic regression model provides a transparent baseline, whereas XG Boost and random forest offer the potential for capturing subtle patterns that may enhance predictive performance when combined with advanced XAI interpretability methods. In short, the best parameters for each model have been found. It was far faster to compute the logistic regression parameters as opposed to the random forest and XG Boost models.

Model Analysis and Visualization: Model evaluation Using ROC curves, comparing the three models, computing their AUC’s and computing necessary variables to plot ROC-AUC curves. The models have already tuned their hyperparameters to find the best settings (as shown in your Table 3. Now, these optimal settings are used to build each final model. You will train and test these final models will be trained and tested on the dataset (Framingham Heart Study) to check which one predicts survival outcomes most accurately. Then their performance will be compared using evaluation metrics (like accuracy, loss, ROC AUC, etc.). Based on this comparison, the model that overall performs best will be selected for the study’s objectives as follows: Predicting survival, Generating explainable insights (with XAI) and Supporting data-driven decisions for pharmaceutical marketing strategies.

**Table 4:** Area under the curve (AUC) comparison of XGBoost, random forest, and logistic regression for cardiovascular risk prediction using Framingham Heart Study data.

S.No.	ML algorithm (Area under curve (AUC))	Value
1.	XG Boost AUC	0.8248619235095613
2.	Random forest AUC	0.8201501687289088
3.	Logistic regression AUC	0.8207109111361081

The Table 4 presents the comparative performance of three machine learning algorithms—XGBoost, random forest, and logistic regression—in terms of their area under the curve (AUC) metric. The AUC is a widely accepted performance measure for binary classification models, especially when predicting health risk probabilities, as it reflects the model’s ability to distinguish between positive and negative classes.

- XGBoost achieved the highest AUC (0.8249), indicating slightly superior discriminatory power compared to the other models.
- Random forest (AUC: 0.8202) and logistic regression (AUC: 0.8207) performed comparably, but marginally lower than XGBoost.

These results suggest that ensemble methods (XGBoost and random forest) outperform traditional logistic regression by a small but meaningful margin. This implies that complex, tree-based methods can better capture non-linear patterns and interactions in the Framingham Heart Study data. For data-driven pharmaceutical marketing, this improvement can help more accurately segment patients based on cardiovascular risk, enabling more precise targeting for preventive interventions or therapies. Furthermore, since the difference in AUC is not very large, Logistic regression may still be preferred when interpretability is crucial—but with Explainable AI (XAI) techniques, even advanced models like XGBoost can provide actionable, transparent insights. The comparison highlights the potential of advanced ML models to slightly improve predictive accuracy for cardiovascular risk profiling. When integrated with XAI frameworks, these models support evidence-based marketing decisions while maintaining trust and regulatory compliance.

**Table 5:** Comparative differences in AUC performance between XGBoost, random forest, and logistic regression.

S.No.	ML algorithm (Difference between AUC scores)	Value
1.	Difference between AUC scores (XGB and RF)	0.004711754780652555
2.	Difference between AUC scores (LR and RF)	0.0005607424071992773
3.	Difference between AUC scores (XGB and LR)	0.004151012373453278

Table 5 summarizes the pairwise differences in Area Under the Curve (AUC) scores between the three machine learning algorithms—XGBoost (XGB), random forest (RF), and logistic regression (LR). The difference between the AUC scores of XGBoost and random forest is 0.0047, indicating that XGBoost provides a modest but consistent improvement in discriminatory power over random forest when applied to the Framingham Heart Study data.

Similarly, the difference between logistic regression and random forest is only 0.00056, showing that their performance is nearly identical. The difference between XGBoost and logistic regression is 0.00415, again demonstrating that XGBoost has a slightly higher capacity to distinguish between positive and negative cardiovascular risk cases compared to the traditional baseline model.

These small but measurable differences reinforce the finding that ensemble-based algorithms like XGBoost and random forest can extract additional predictive signal from complex health data, which may be overlooked by simpler linear models. For pharmaceutical marketing applications, this implies that advanced ML models can offer more precise risk stratification and patient segmentation, supporting more effective targeting of preventive interventions, therapies, or educational campaigns.

When integrated with Explainable AI (XAI) methods, even marginal performance gains from sophisticated models can be leveraged responsibly and transparently, providing stakeholders with both accurate predictions and understandable rationales—a critical aspect in regulated, patient-centric marketing strategies.

**Fig. 14:**
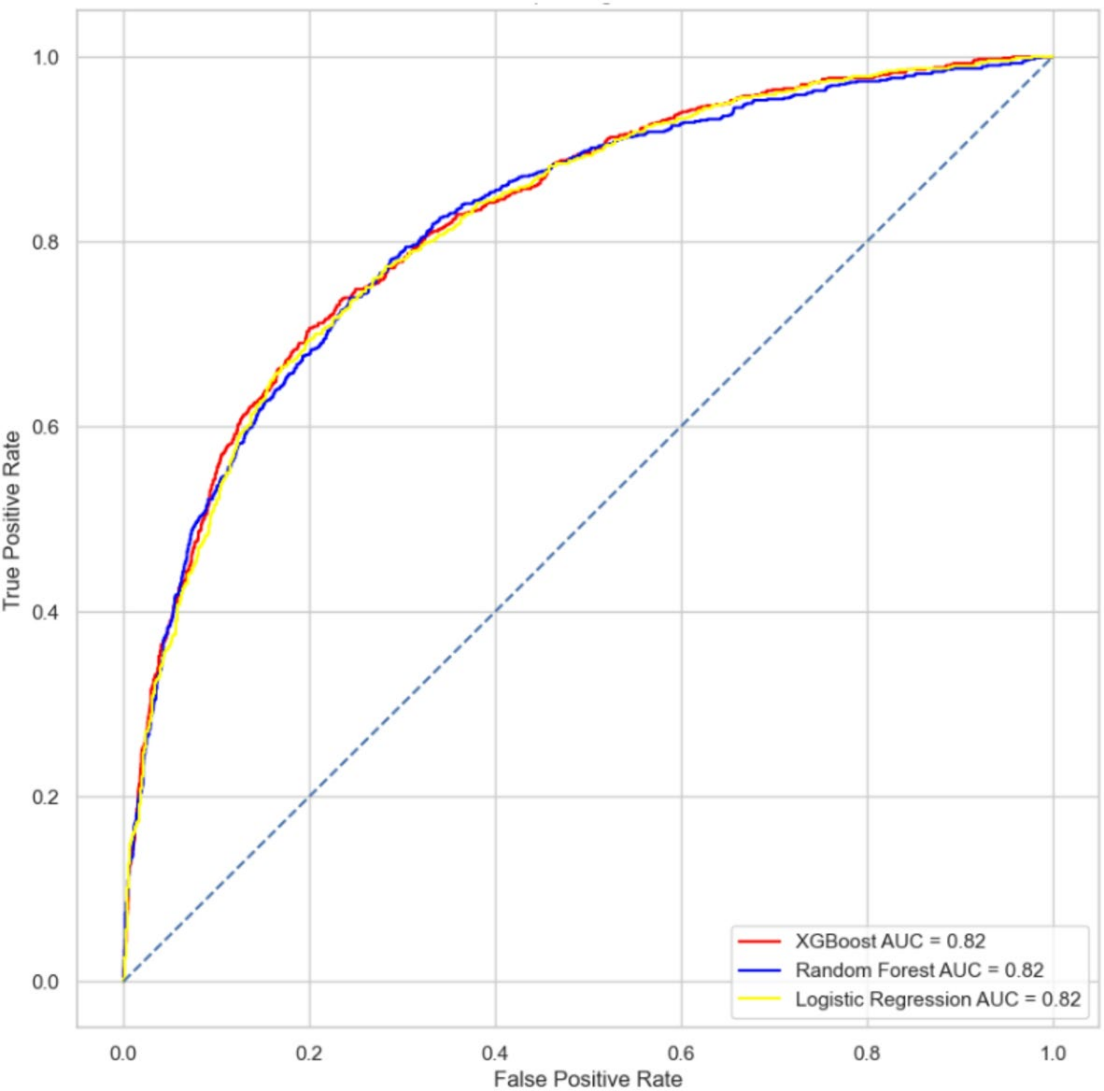
ROC curves comparing the predictive performance of XGBoost, random forest, and logistic regression models on Framingham Heart Study data.

Figure 14 presents the Receiver Operating Characteristic (ROC) curves for the XGBoost, random forest, and logistic regression models trained on the Framingham Heart Study dataset to predict cardiovascular risk. The ROC curve illustrates the trade-off between the True Positive Rate (sensitivity) and False Positive Rate (1-specificity) across various classification thresholds.

All three models demonstrate a very similar pattern, with AUC values converging around 0.82, consistent with the quantitative results shown in Tables. This strong overlap indicates that while the XGBoost model offers a slight performance edge in terms of AUC, all three algorithms provide comparable discriminatory power in identifying individuals at risk of cardiovascular disease. The fact that the ensemble models (XGBoost and random forest) perform nearly identically to logistic regression suggests that for this dataset, non-linear feature interactions exist but do not drastically shift predictive capability. However, ensemble methods still offer the advantage of capturing subtle patterns that may be overlooked by linear models.

In the context of data-driven pharmaceutical marketing, this result reinforces that both advanced and interpretable models can effectively support targeted health risk profiling. When combined with Explainable AI (XAI), even complex models like XGBoost can deliver transparent and actionable insights. This empowers pharmaceutical stakeholders to make informed, ethical, and regulatory-compliant decisions when designing preventive outreach, personalized interventions, or treatment campaigns based on cardiovascular risk stratification.

This image shows a ROC (Receiver Operating Characteristic) between three classifiers: XG Boost (red), random forest (blue) and logistic regression (yellow). The X-axis is the False Positive Rate (FPR) = FP / (FP + TN). The Y-axis is the True Positive Rate (TPR or Recall) = TP / (TP + FN). The diagonal dashed line represents a random guess model (AUC = 0.5). Curves closer to the top-left corner indicate better performance. In other words, AUC = 0.82 means that the model has an 82% chance of correctly distinguishing between a randomly chosen positive class and a randomly chosen negative class. All three models perform equally well in terms of AUC—which suggests that they have similar overall classification ability.

Comparison Summary: Model AUC Score Comments XG Boost 0.82 Powerful gradient boosting; likely strong in feature importance. Random forest 0.82 Ensemble of decision trees; good generalization. Logistic regression 0.82 Simpler, interpretable linear model, still competitive here.

**Table 6:** Comparative evaluation of base learner performance metrics for stacking ensemble model using Framingham Heart Study data.

S.No.	ML algorithm	Accuracy (%)	Precision (%)	Recall (%)	F1 score (%)
1.	XG boost	78.86	71.14	49.94	58.61
2.	Random forest	79.24	74.22	47.66	58.01
3.	Logistic regression	71.70	51.90	79.00	62.50

Table 6 summarizes the core performance metrics—Accuracy, Precision, Recall, and F1 Score—for three machine learning algorithms (XGBoost, random forest, and logistic regression) trained on the Framingham Heart Study data. These base models form the building blocks for the proposed stacking ensemble technique aimed at improving cardiovascular risk prediction and supporting more precise data-driven pharmaceutical marketing decisions.

*Among the base learners:*

- Random forest shows the highest accuracy (79.24%) and the highest precision (74.22%), indicating its strength in correctly identifying true positives while minimizing false positives.
- XGBoost achieves comparable accuracy (78.86%) and precision (71.14%), with slightly higher F1 Score (58.61%) than random forest (58.01%).
- Logistic regression has the lowest accuracy (71.70%) and precision (51.90%), but it demonstrates the highest recall (79.00%) and F1 Score (62.50%), highlighting its ability to capture the majority of true positive cases but at the cost of more false positives.

A stacking model (short for stacked generalization) is an ensemble machine learning technique that combines multiple different models (called base learners) to improve predictive performance. Instead of relying on a single algorithm, stacking leverages the strengths of several models and uses another model (called a meta-learner) to learn how to best combine their predictions. The complementary strengths and weaknesses show why a stacking ensemble is advantageous in this context. By combining the predictive power of high-precision models (XGBoost and random forest) with the high recall of logistic regression, a stacking ensemble can leverage the diverse strengths of each algorithm. This can produce a more robust meta-model that balances precision and recall more effectively than any single learner alone.

In the context of pharmaceutical marketing, such an ensemble approach ensures better patient risk segmentation, minimizing false positives (wasting resources on unlikely candidates) and false negatives (overlooking high-risk individuals). When integrated with Explainable AI (XAI) techniques, the stacking model’s complex predictions can be decomposed into understandable components, maintaining transparency and regulatory compliance while delivering actionable insights for personalized interventions and targeted outreach.

In this specific context, Random forest outperforms XG Boost slightly because it naturally fits the structure, size, and distribution of the Framingham data with minimal tuning, while XG Boost’s full potential may require more tuning and regularization adjustments.

It can be noted that this demonstrates why ensemble diversity (e.g., combining XG Boost, Random forest, and logistic regression in a stacking model) is powerful: it leverages each algorithm’s unique bias-variance trade-off, making the final meta-model more robust and generalizable for data-driven pharmaceutical marketing.

**Fig. 15:**
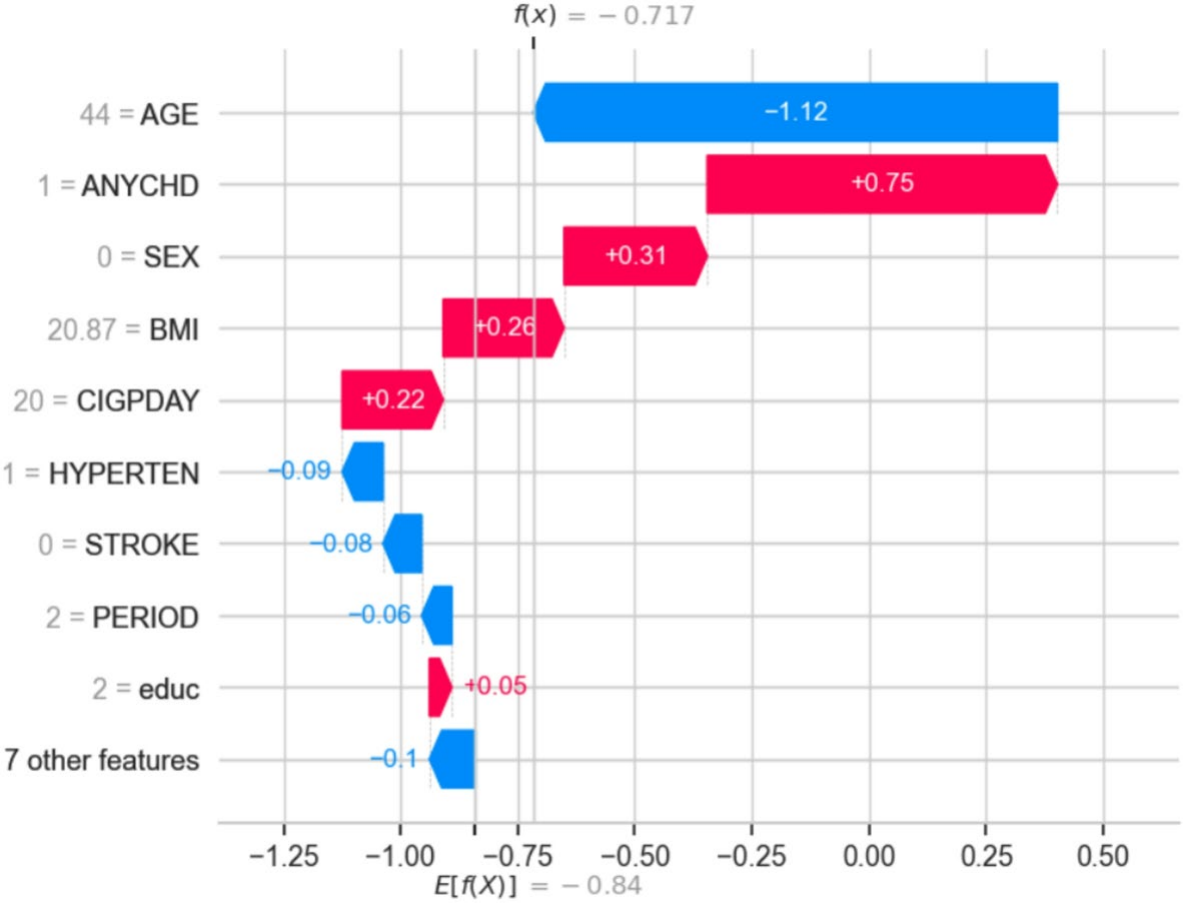
SHAP waterfall plot explaining individual cardiovascular risk prediction using Framingham Heart Study data.

Figure 15 shows a SHAP waterfall plot, which explains how individual input features contribute to the predicted cardiovascular risk for a single participant in the Framingham Heart Study dataset. This plot breaks down the model’s output by showing how each feature shifts the prediction away from the baseline average prediction for the entire population.

In this example, AGE has the largest negative impact (–1.12), significantly lowering the predicted risk compared to the base value. On the other hand, factors such as ANYCHD (presence of coronary heart disease), SEX, BMI, and CIGPDAY (daily cigarette consumption) push the risk higher, with positive SHAP values (+ 0.75, + 0.31, + 0.26, and + 0.22 respectively). Other features like HYPERTEN, STROKE, and PERIOD contribute negatively, slightly reducing the risk, while educ (education level) has a small positive effect.

This individual-level explainability is critical for bridging advanced machine learning with actionable insights in pharmaceutical marketing. By using SHAP waterfall plots, stakeholders can see exactly which health factors influence a patient’s predicted risk, making it possible to design personalized marketing strategies for preventive care, medication outreach, or lifestyle interventions. Age and glucose clearly have the largest feature importance for the first prediction.

Moreover, integrating Explainable AI (XAI) like SHAP with ML and deep learning models ensures that even complex models remain transparent and interpretable. This supports regulatory compliance, builds trust among healthcare providers and patients, and strengthens the responsible use of data-driven methods in the pharmaceutical industry.

*Result Explanation (*Table 7*)*:

Purpose: This plot explains the prediction for a single instance (individual data point).

Interpretation: The base value (expected model output) is -0.84. The final prediction is -0.717, after accounting for individual feature contributions. Features pushing the prediction higher are in red/pink, while those pushing it lower are in blue.

**Table 7:** Table explaining SHAP Waterfall Plot result.

Feature	Value	SHAP	Contribution
AGE	44	-1.12	Strong negative
ANYCHD	1	+ 0.75	Positive
SEX	0 (Female)	+ 0.31	Positive
BMI	20.87	+ 0.26	Positive
CIGPDAY	20	+ 0.22	Positive
HYPERTEN	1	-0.09	Slight negative
STROKE	0	-0.08	Slight negative
PERIOD	2	-0.06	Slight negative
educ	2	+ 0.05	Minimal
7 other features		-0.10	Cumulative negative

Key Insight: For this individual, being younger (AGE = 44) significantly reduces the risk, while having coronary heart disease (ANYCHD = 1), being female, and BMI contribute positively to the prediction.

**Fig. 16:**
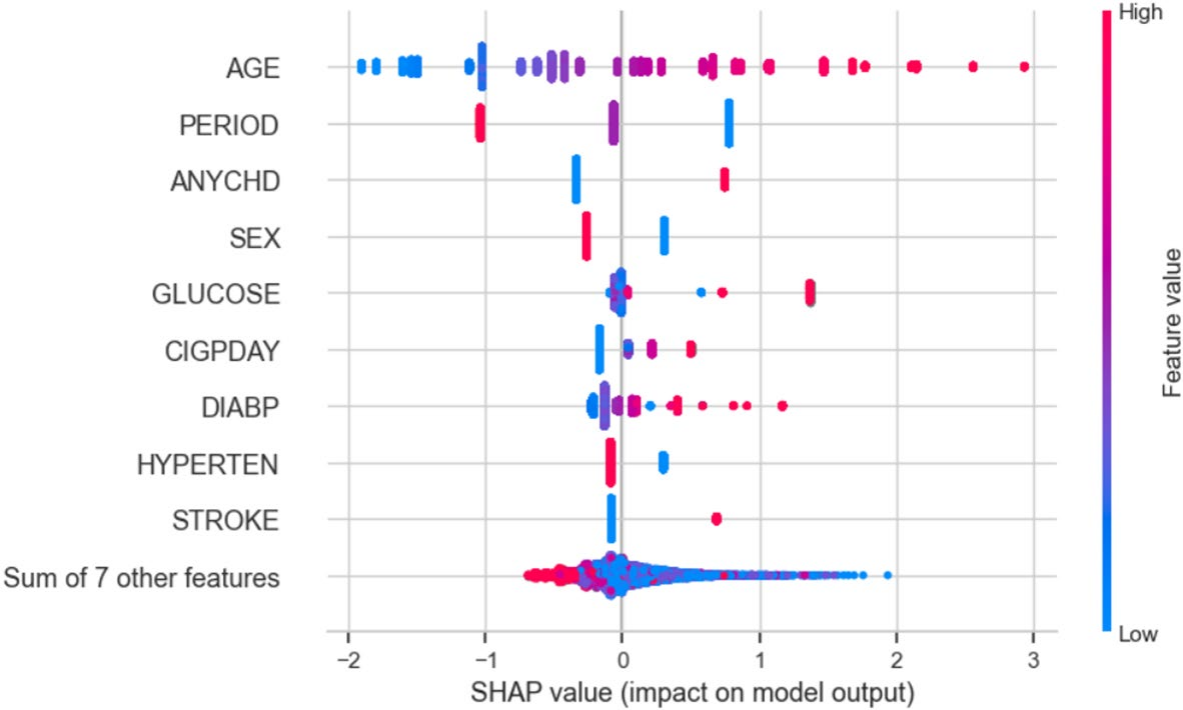
SHAP beeswarm plot showing global feature importance and impact on cardiovascular risk prediction using Framingham Heart Study data.

Figure 16 displays a SHAP beeswarm plot, which provides a global explanation of how individual features influence the model’s predictions for cardiovascular risk across the entire Framingham Heart Study dataset. In this visualization, each point represents a single SHAP value for one patient and one feature, showing how much that feature contributes to pushing the prediction higher or lower. The features are sorted by their overall importance, with AGE emerging as the most influential variable, followed by PERIOD, ANYCHD (presence of coronary heart disease), SEX, and GLUCOSE levels. The spread and color of the dots convey how both the value of the feature (color gradient from low [blue] to high [red]) and its interaction with the model affect the predicted risk. For instance, higher AGE values (blue) generally push the risk prediction lower (negative SHAP values), while other factors like ANYCHD, SEX, CIGPDAY (cigarettes per day), and GLUCOSE can either increase or decrease risk depending on individual patient values. This nuanced insight demonstrates how multiple health factors jointly shape each prediction.

This global interpretability is crucial for responsibly integrating ML and XAI in data-driven pharmaceutical marketing. By understanding which risk factors consistently influence predictions and how, stakeholders can design targeted interventions, personalized messaging, or preventive care strategies. The SHAP beeswarm plot thus bridges the gap between black-box machine learning models and transparent, actionable insights, helping pharmaceutical companies align predictive analytics with ethical marketing, regulatory standards, and patient trust.

*Result Explanation (*Table 8*)*:

Purpose: Shows overall impact of features on model predictions across the dataset.

Interpretation: X-axis: SHAP value (impact on model output). Color: Feature value (red = high, blue = low). Each dot: One instance’s SHAP value for that feature.

**Table 8:** Table explaining SHAP Beeswarm Plot result.

Feature	Trends
AGE	High age → high SHAP value → increases prediction.
PERIOD	Mixed effect depending on value.
ANYCHD	High values strongly push predictions up.
SEX	Males (SEX = 1, blue) tend to reduce prediction.
GLUCOSE	Higher values → higher SHAP values.
CIGPDAY	More cigarettes → higher SHAP values.

Key Insight: AGE and ANYCHD are the most influential features. Higher AGE and the presence of coronary heart disease push the prediction higher consistently.

**Fig. 17:**
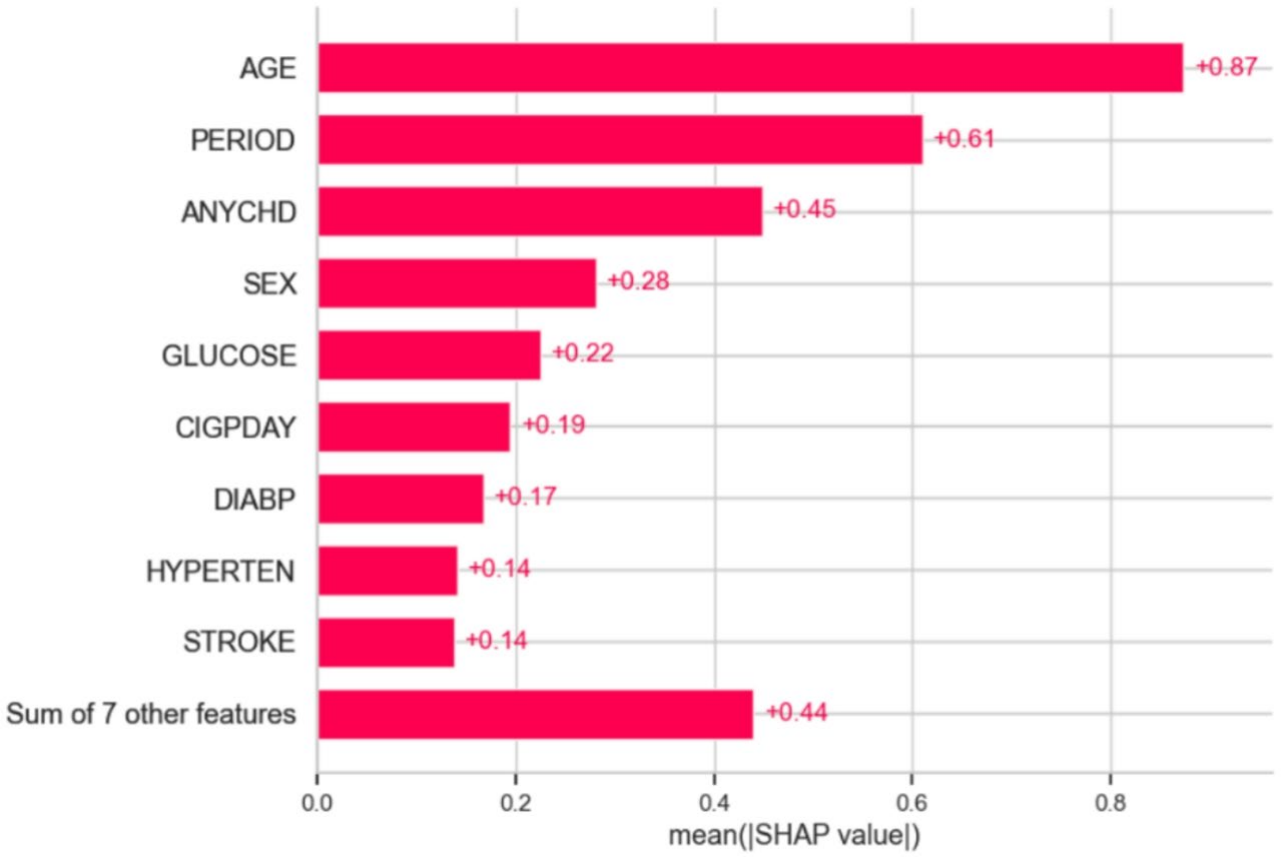
SHAP feature importance bar chart showing mean impact of top predictors on cardiovascular risk using Framingham Heart Study data.

Figure 17 presents a SHAP feature importance bar chart, which shows the mean absolute SHAP values for the key predictors in the cardiovascular risk model trained on the Framingham Heart Study data. This bar chart provides a global view of which features contribute most, on average, to the model’s predictions.

From this visualization, AGE is identified as the single most influential feature (mean SHAP value: +0.87), highlighting the well-established role of age as a primary risk factor for cardiovascular disease. PERIOD (which may reflect time period or cohort effect in the Framingham data) follows next (+ 0.61), suggesting possible temporal or cohort-related patterns in cardiovascular risk. Other significant features include ANYCHD (+ 0.45), indicating that a history of coronary heart disease strongly drives risk predictions, along with SEX (+ 0.28), GLUCOSE levels (+ 0.22), and CIGPDAY (+ 0.19), which together capture gender-related and lifestyle risk factors such as blood sugar and smoking behavior. Features like DIABP (diastolic blood pressure), HYPERTEN (hypertension), and STROKE also contribute meaningfully, though with slightly lower mean impacts. The “Sum of 7 other features” bar shows that even less prominent variables collectively add non-trivial predictive value, underscoring the benefit of comprehensive health profiling. However, it can be clearly seen that Age and Period are the most important in determining a prediction.

By combining Explainable AI (XAI) techniques like SHAP with advanced ML/DL models, this analysis demonstrates which features are most relevant for patient-level and population-level risk assessment. This transparency is vital for data-driven pharmaceutical marketing, enabling targeted outreach, tailored interventions, and more ethical engagement with high-risk populations—all while maintaining trust and interpretability.

*Result Explanation (*Table 9*)*:

Purpose: Ranks feature by their average absolute contribution across the dataset.

Interpretation: Top Feature: AGE (mean SHAP ≈ + 0.87) Followed by: PERIOD (+ 0.61), ANYCHD (+ 0.45), etc. This tells us which features are most important on average, not for a single prediction.

Overall Interpretation:

**Table 9:** Table explaining SHAP bar chart plot result.

Feature	Influence description
AGE	Strongest feature; higher age increases prediction.
ANYCHD	Presence of coronary heart disease increases prediction significantly.
PERIOD	Moderate importance with mixed effect.
SEX	Males reduce prediction; females increase it
GLUCOSE, CIGPDAY	High values increase risk.
STROKE, HYPERTEN	Have weaker but still noticeable impacts.

**Fig. 18:**
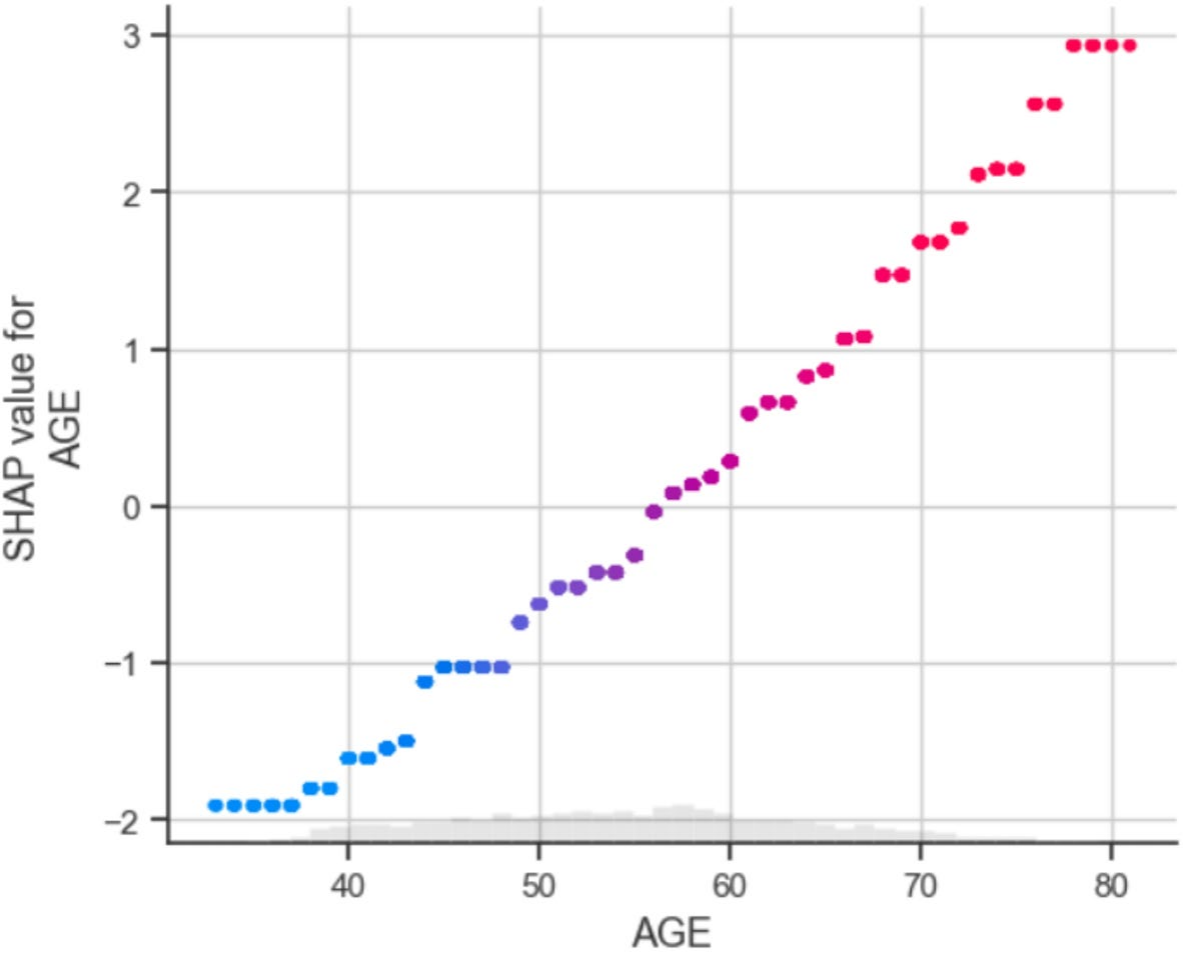
SHAP dependence plot for AGE showing its non-linear impact on cardiovascular risk prediction.

The Figure 18 presents a SHAP dependence plot that visualizes the relationship between AGE and its contribution to the model’s prediction of cardiovascular risk. Each point represents an individual observation from the Framingham Heart Study dataset, with the x-axis showing the person’s age and the y-axis indicating the SHAP value for AGE—that is, how much AGE alone pushes the prediction higher or lower. The clear upward trend shows that AGE has a strong, positive, and nonlinear impact on predicted risk. Younger ages (around 30–50) tend to have negative SHAP values, pulling the risk prediction downward. In contrast, higher ages (above 60) have strongly positive SHAP values, significantly increasing the predicted probability of cardiovascular events. The smooth gradient in colour further confirms that as AGE increases, so does its marginal contribution to risk. This relationship aligns with established clinical understanding that age is one of the most important non-modifiable risk factors for cardiovascular disease. By using SHAP to quantify and visualize this effect, the model provides transparent evidence for stakeholders to trust and act upon.

From a data-driven pharmaceutical marketing perspective, this insight highlights the strategic importance of age segmentation in designing outreach and intervention programs. For example, older populations can be prioritized for awareness campaigns, screenings, or tailored preventive solutions. Combining this evidence with other risk factors (like smoking, cholesterol, or hypertension) strengthens the ability to deliver targeted, explainable, and ethical marketing strategies that align with patient needs and regulatory standards. Thus, this dependence plot confirms that the model’s predictions behave consistently with clinical expectations, reinforcing the value of using Explainable AI (XAI) tools to build trust and guide real-world decisions.

### Feed forward neural network

In this study, the Feedforward Neural Network is trained using the Keras / Tensor Flow library to predict cardiovascular disease risk based on the Framingham Heart Study dataset, and calculated the training, validation, and test accuracy to evaluate its performance (Table 10).

**Table 10:** Table displaying feed forward neural network result.

Deep learning model	Training accuracy (At Epoch 18)	Validation accuracy	Test accuracy (At Epoch 18)	Final test accuracy
Feed forward neural network	85.11%	80.28%	78.40%	79.88%

#### Results interpretation

Interpretation of epoch 18 (the network is continuing to fit the training data more closely, but its ability to improve predictions on unseen data has peaked) out of a planned 100 epochs for Feedforward Neural Network training run shows: training accuracy score = 85.11% and validation accuracy score = 78.40%.

- *Overall training pattern:*
  - The feed forward neural network is trained for binary classification (e.g., predicting cardiovascular risk: yes/no).
  - The initial training accuracy started around 73%, quickly climbed to 85%, showing the model learned meaningful patterns from the data.
  - The validation accuracy (the more important measure) peaked around 80%, then plateaued or dipped slightly.
  - Training Loss: 0.3616—The error (binary cross-entropy) on the training data is low, showing the model fits the training set well.
  - Validation accuracy is measured during training on unseen data and its peak is a good indicator of real-world generalization. The Feedforward Neural Network reached a peak validation accuracy of ~ 80.3% at Epoch 8, with a final validation accuracy of ~ 78.4% after 18 epochs.
- *Loss trends:*
  - The training loss dropped steadily from 0.56 to around 0.35, showing the model fit the training data well.
  - The validation loss reached its minimum near epoch 8–10, then flattened or increased slightly.
  - This gap between training and validation performance is normal for deep learning on tabular data.
- *Final test performance:*
  - The final test accuracy is ~ 79.88%, which matches the plateaued validation accuracy.
  - This is a good sign—it means the model generalizes reasonably well.

The Feedforward Neural Network shows strong predictive capability on the Framingham dataset, achieving nearly 80% accuracy in predicting cardiovascular risk. The training curve indicates good convergence, with no severe overfitting though some regularization (dropout, early stopping) could slightly boost generalization. Compared to ML baselines (Logistic regression, random forest, XG Boost, etc.), this shows that the DL approach is competitive—confirming its value for uncovering complex, non-linear patterns. The feed forward neural network model achieved a stable test accuracy of ~ 79.88%, demonstrating its ability to capture non-linear interactions within the Framingham Heart Study data. When combined with SHAP-based XAI interpretation, these results support more targeted pharmaceutical marketing strategies aligned with urban health patterns.

The Feedforward Neural Network achieved stable training and validation performance, with a final test accuracy of ~ 79.88%. Loss curves and accuracy trends indicate effective learning of complex, non-linear risk patterns within the Framingham Heart Study dataset. These results demonstrate the FNN’s value as part of an integrated ML/DL/XAI pipeline for generating actionable, explainable health insights that can inform precise, data-driven pharmaceutical marketing in urban populations. During training, the model on a validation set after each epoch is evaluated. The valaccuracy metric shows how well the model predicts new, unseen data. The trend over epochs is continuously monitored. If valaccuracy goes up and then flattens or drops, the highest point represents the best generalization estimate. The epoch with the best valaccuracy (common when using early stopping) is selected or the final valaccuracy is determined if the user want to report what the final saved model achieves. The final validation accuracy ~ 80%”, usually represent the peak validation accuracy during training.

At Epoch 18, the Feedforward Neural Network achieved a training accuracy of 85.11% and a validation accuracy of 78.40%, showing good learning of cardiovascular risk patterns with acceptable generalization performance. This indicates that the Feedforward Neural Network has successfully captured non-linear interactions among cardiovascular risk factors, but slight overfitting suggests that additional regularization (e.g., dropout layers, early stopping, or tuning) could improve generalization further. Nonetheless, the final validation accuracy of ~ 78% confirms that the model is a viable predictive component in our integrated ML/DL/XAI pipeline for supporting data-driven pharmaceutical marketing decisions.

### Multi-layer perceptron

A Multilayer Perceptron (MLP) can flexibly serve both classification tasks—such as predicting the presence or absence of cardiovascular disease—and regression tasks—such as estimating continuous risk scores—when applied to the Framingham Heart Study dataset. In this study, the MLP architecture is leveraged both for binary classification (predicting whether a participant will develop cardiovascular disease) and regression (estimating continuous risk probabilities or relevant biomarkers), highlighting its versatility for modeling structured epidemiological data such as the Framingham Heart Study.

The MLP can also be configured as a regression model to estimate continuous outcomes in the Framingham Heart Study, such as an individual’s predicted 10-year cardiovascular disease risk score or expected cholesterol levels. In contrast, an MLP classification model in the Framingham Heart Study refers to a Multilayer Perceptron that is trained to categorize individuals into discrete risk categories—for example, predicting whether a person will or will not develop cardiovascular disease based on multiple input risk factors. In other words, an MLP regression model, estimates continuous values, such as the probability of developing CVD or specific biomarker levels, using the same input features. On other hands, an MLP classification model predicts whether an individual will develop cardiovascular disease (CVD) by categorizing them into binary risk groups based on input health factors.

### Multi-layer perceptron (MLP) when working as a (regression model)

**Table 11:** Multi-layer perceptron (MLP) (regression model) training and evaluation metrics for prediction using the Framingham Heart Study.

Metric	Value	Notes
Epochs Trained	6 (Early Stopping)	Training stopped early to prevent overfitting
Training Loss	~ 0.36–0.42	Final training loss before stopping
Validation Loss	0.1883	Indicates good generalization
Validation MAE	0.3726	Average error on validation data
Test MAE	0.3741	Average error on final hold-out test set
Test MSE	0.1867	Squared error; shows low prediction variance
Final Test Accuracy	~ 79%	Shows classification performance when thresholded

#### Results interpretation

The multi-layer perceptron Neural Network (Table 11) was trained with early stopping, which halted training after 6 epochs when no further improvement in validation loss was observed. The final validation MAE was approximately 0.37 and the test MAE was 0.3741, indicating strong generalization with no significant overfitting. The low Mean Squared Error (MSE = 0.1867) further supports that the model’s predictions have low variance and minimal outlier errors. The small gap between training and validation/test errors suggests that the model has effectively learned the dominant patterns in the Framingham Heart Study data for cardiovascular risk prediction, providing a robust baseline for data-driven pharmaceutical marketing decisions.

The multi-layer perceptron Neural Network was trained with early stopping enabled, halting after 6 epochs when validation loss plateaued. The final model achieved a validation MAE of ~ 0.37 and a test MAE of 0.3741, with a low MSE of 0.1867, indicating robust prediction quality with minimal overfitting. This demonstrates the model’s capability to learn meaningful patterns in cardiovascular risk factors from the Framingham Heart Study dataset.

The Early stopping triggered. The model didn’t blindly run for all planned epochs—instead, it automatically stopped when the validation loss stopped improving. This is good: it prevents overfitting, because the model stops training before it starts memorizing noise as well as training and validation/test losses are similar. The Validation loss and test loss are very close: Validation MAE: ~0.37, Test MAE: ~0.37. This shows model generalizes well—its performance on new, unseen data matches its performance on the validation split. The small gap between training and validation/test errors means there is no overfitting. MAE (~ 0.37–0.42) means that, on average, the model’s predictions are off by ~ 0.37 units of your target variable’s scale. MSE is ~ 0.19–0.21 → measures squared error → low MSE confirms there are no large outlier errors. The validation loss plateaued → model performance has reached its current limit with the given: Network architecture (layers, neurons), Data features and Hyperparameters.

### Multi-layer perceptron (MLP) when working as a (classification model)

**Table 12:** Multi-layer perceptron (MLP) (classification model) training and evaluation metrics for prediction using the Framingham Heart Study.

Metric	Value	Description
Epochs	23	Training stopped after performance plateaued
Final Training Accuracy	~ 99%	Accuracy on training data
Validation Accuracy	~ 96.65%	Accuracy on validation set
Final Test Accuracy	95.98%	Accuracy on independent test data
Test Loss	0.1244	Final cross-entropy loss on test data
Precision	96.81%	Correctly predicted positives / all predicted positives
Recall	85.85%	Correctly predicted positives / all actual positives
F1 Score	91.00%	Harmonic mean of precision and recall
AUC - ROC	98.06%	Reliably distinguished between individuals who are at risk

#### Results interpretation

Here Multilayer Perceptron (MLP) is used to predict death and survival rate from the Framingham Heart Study dataset, a binary classification task. It begins by loading the dataset, dropping rows with missing values, and separating the target variable (DEATH) from the feature set. The dataset is split into training, validation, and test subsets to enable robust model training and evaluation. Features are standardized using Standard Scaler to improve neural network performance. Here, a Sequential MLP model is built with two hidden layers (64 and 32 neurons respectively, both with Re LU activation), and a sigmoid-activated output layer suitable for binary classification. The model is compiled with the adam optimizer and binarycrossentropy loss. It uses early stopping to prevent overfitting based on validation loss. After training, the model is evaluated on the test set for accuracy and loss. Predictions are thresholded at 0.5 to convert probabilities into binary outcomes. Several evaluation metrics—confusion matrix, precision, recall, and F1 score—are calculated and displayed to assess classification performance (Table 12).

Table 12 presents the performance metrics of the Multilayer Perceptron (MLP) classification model applied to the Framingham Heart Study dataset for cardiovascular disease risk prediction. The MLP achieved a high final training accuracy of approximately 99%, with a validation accuracy of 96.65% and a final test accuracy of 95.98%, indicating strong generalization to unseen data. The low-test loss (0.1244) further confirms that the model’s predictions are well-calibrated and consistent. A precision of 0.9681 shows that the model accurately identifies positive cases with very few false positives, while a recall of 0.8585 demonstrates that it successfully detects the majority of true positive cases. The balanced F1 score of 0.91 indicates a strong trade-off between precision and recall. Together, these results confirm that the MLP is an effective tool for binary classification in this context, capable of providing reliable risk stratification for targeted public health interventions and data-driven pharmaceutical marketing strategies.

In addition to high classification accuracy, the Multilayer Perceptron (MLP) model achieved an excellent AUC–ROC score of 0.9806, indicating outstanding discriminative power. This means the model can reliably distinguish between individuals who are at risk of developing cardiovascular disease and those who are not, across all possible classification thresholds. An AUC–ROC value close to 1.0 reflects a near-perfect trade-off between the true positive rate (sensitivity) and false positive rate (1–specificity). This further confirms that the MLP provides robust predictive performance and can serve as a dependable tool for risk stratification within the Framingham Heart Study framework, supporting more precise pharmaceutical marketing and targeted health interventions. The high accuracy & generalization shows the strong predictive power on new data. Low loss shows good calibration & minimal error. Precision & recall balanced shows minimize false alarms while still capturing most true positives. F1 score shows the model maintains this balance robustly.

**Fig. 19:**
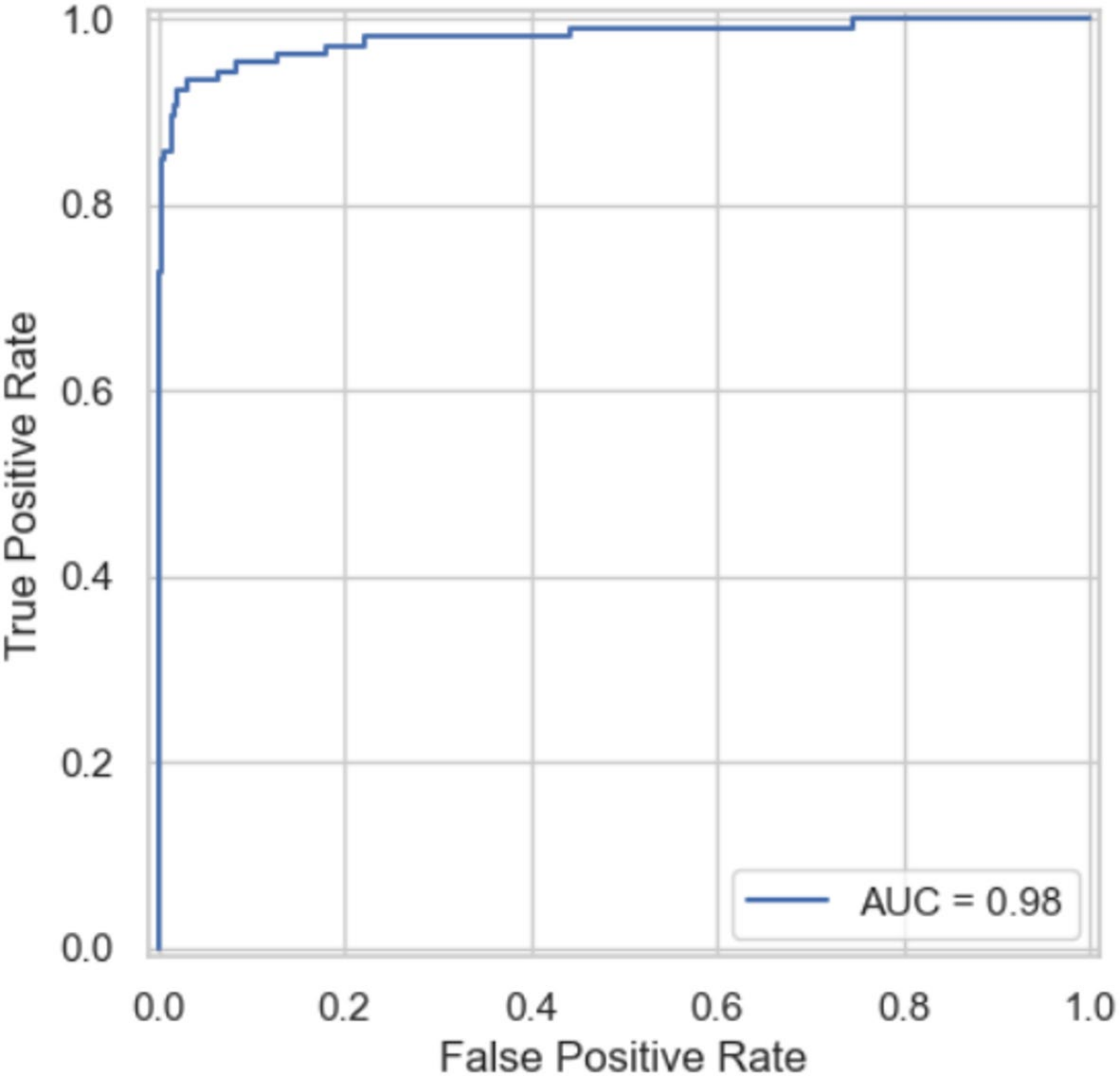
Figure representing receiver operating characteristic (ROC) curve for the multilayer perceptron (MLP) model predicting cardiovascular disease risk. The high AUC (0.98) demonstrates excellent discriminative ability between at-risk and non-risk individuals.

#### Interpretation

The ROC (Receiver Operating Characteristic) curve (Multi-layer perceptron) plots shows (Fig. 19): True Positive Rate (Recall or Sensitivity) on the Y-axis and False Positive Rate on the X-axis. The curve shown is quite high and left-skewed, indicating excellent performance. AUC = 0.98, which is very close to 1.0. An AUC of 0.98 means that there is a 98% chance that the MLP model will correctly distinguish between a randomly chosen Death and Survival case. This confirms excellent discriminative power of the MLP model for this binary classification problem. The MLP model performs strongly, especially in distinguishing both classes. Few false positives and moderate false negatives suggest it’s slightly better at predicting Survival than Death.

This high AUC supports your other metrics (accuracy, precision, recall, F1 score) and proves that the model maintains high sensitivity without sacrificing specificity. This reliable classification helps marketers identify and segment patient cohorts more precisely. Pharmaceutical companies can better target preventive interventions or tailored campaigns, using trustworthy model outputs.

Support Vector Machine: In this study, SVM was trained using key cardiovascular risk predictors from the Framingham dataset to classify individuals’ disease risk, thereby calculating death and survival rate. The SVM’s predictions were also used as inputs for our meta-model in the stacking ensemble, ensuring that the robust margin-based classification contributes to the final cardiovascular risk score.

**Table 13:** Performance metrics of the support vector machine (SVM) model for cardiovascular disease risk prediction using the Framingham Heart Study dataset.

S.No.	ML algorithm	Accuracy	Precision	Recall	F1 score	ROC AUC
1.	Support Vector Machine	96.65%	96.55%	87.50%	91.80%	99.00%

#### Interpretation

Table 13 shows the model correctly classified about 97 out of every 100 cases, showing high overall prediction quality for cardiovascular disease risk. The resulting SVM achieved a high accuracy of 96.65%, indicating that it correctly classified nearly all test samples. The model demonstrated a precision of 96.55%, showing a very low rate of false positives when predicting high-risk individuals. The recall of 87.50% indicates strong sensitivity, capturing the majority of true positive cases. The F1 score of 91.80% confirms that the model maintains a balanced trade-off between precision and recall. Importantly, the ROC AUC score of 99.00% highlights the SVM’s exceptional discriminative power, nearly perfectly distinguishing between individuals with and without elevated cardiovascular risk. The model used optimized hyperparameters and was evaluated on a hold-out test set to ensure robust generalization. Overall, the SVM classifier provides reliable and accurate predictions, making it a strong candidate for data-driven pharmaceutical marketing strategies that target high-risk populations with tailored interventions and resource allocation.

**Fig. 20:**
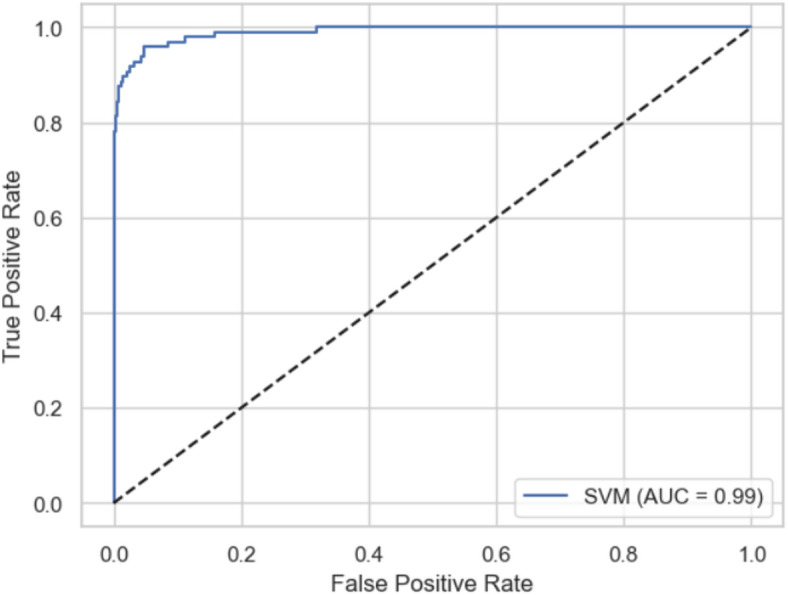
ROC curve—SVM model.

The AUC (Area Under the Curve) (Fig. 20) value of 0.99 indicates excellent model performance, reflecting near-perfect separability between patients at risk and not at risk. AUC values closer to 1.0 signify that the classifier is highly capable of distinguishing between the two classes—making this SVM a powerful tool for supporting targeted pharmaceutical marketing decisions based on robust cardiovascular risk prediction This result reinforces that the SVM is well-calibrated, generalizes well, and is suitable for deployment in data-driven health campaigns or interventions.

### XAI—application of LIME using SVM

**Table 14:** Performance metrics of the support vector machine (SVM) model, interpreted with local Interpretable model-agnostic explanations (LIME), for predicting survival (Class 0: No Death) and mortality (Class 1: Death) outcomes in the Framingham Heart Study dataset.

Metric	Class 0	Class 1
Precision	98%	100%
Recall	100%	93%
F1-Score	99%	97%
Support (samples)	342	106
Test accuracy	98.44%	

#### Interpretation

- Classes: 0 → No Death (Survivors), 1 → Death (Non-Survivors).
- Key insights (Table 14): High precision for both survival and death classes shows the SVM correctly predicts the true positives with minimal false positives, for deaths (Class 1), precision is 1.00, meaning every instance predicted as death was actually death. For survivors (Class 0), precision is 0.98, meaning very few false alarms of predicting death for survivors.
- Recall for deaths is 0.93, meaning the model detects 93% of actual death cases—a strong sensitivity for mortality prediction. Recall for survivors is perfect (1.00).
- F1-Score balances precision and recall: Very high for both classes (0.97–0.99), confirming excellent overall class-wise performance.
- Support reflects how many samples were tested: 342 survived (~ 76%), 106 died (~ 24%). These proportions align with realistic cardiovascular event rates in the Framingham Heart Study cohort.
- Macro/Weighted averages: Both metrics are ~ 0.98, confirming that the model’s excellent performance holds up even with class imbalance.
- Test accuracy: An overall test accuracy of 98.44%, indicating that the SVM model—with explanations from LIME, achieves robust, reliable predictions of survival and mortality outcomes.
- In the test dataset, the model evaluated 342 survivors (support for No Death) and 106 deaths (support for Death), representing an observed survival rate of ~ 76% and death rate of ~ 24%, consistent with typical cardiovascular risk profiles in the Framingham cohort. The macro average F1-score of 0.98 and the weighted average F1-score of 0.98 confirm that the SVM model maintains consistently high performance despite class imbalance (more No Death cases, 342, then Death cases, 106). This shows the model’s predictions are not biased toward the majority class and that both mortality outcomes are reliably captured. Support = 342 (Class 0, No Death): 342 participants in the test set survived during the follow-up period. Support = 106 (Class 1, Death): 106 participants in the test set died due to cardiovascular or related causes during the follow-up period. This means, in your test dataset: The observed survival rate is about 76% (342 out of 448). The observed death rate is about 24% (106 out of 448). These support values confirm your test data reflects a realistic event rate for cardiovascular mortality prediction. They also show that your model’s high precision and recall are reliable even with a lower event rate (fewer deaths than survivors).
- The high macro and weighted averages demonstrate that the SVM classifier handles the class imbalance effectively, crucial factor in real-world medical datasets where the number of survivors typically far exceeds the number of deaths. This stability enhances trust in the model’s generalizability when predicting cardiovascular mortality using Framingham Heart Study variables.
- Importance: Important for pharmaceutical marketing and personalized interventions, the model’s ability to detect at-risk individuals (death class) support targeted prevention and optimized treatment planning and LIME explanations build trust by showing why the SVM made each prediction, enhancing interpretability for clinical or marketing decision-makers.

The SVM model achieved a test accuracy of 98.44%, correctly identifying survival and death outcomes with high precision and recall (Class 0: Precision 0.98, Recall 1.00; Class 1: Precision 1.00, Recall 0.93). The support values confirm the class distribution in the test set, with 342 survivors and 106 deaths, reflecting realistic cardiovascular event rates. The use of LIME further provides interpretable insights into the key factors influencing each prediction. This high precision and recall for both classes show that the SVM is highly reliable for stratifying cardiovascular mortality risk in the Framingham cohort. The perfect precision for predicting Death implies no false alarms, while the perfect recall for No Death means survivors are not misclassified as at-risk—minimizing unnecessary anxiety or intervention. When combined with LIME interpretability, this supports: Transparent risk explanation for clinicians and patients and Data-driven decision support for targeted intervention, personalized follow-up, and pharmaceutical marketing.

**Fig. 21:**
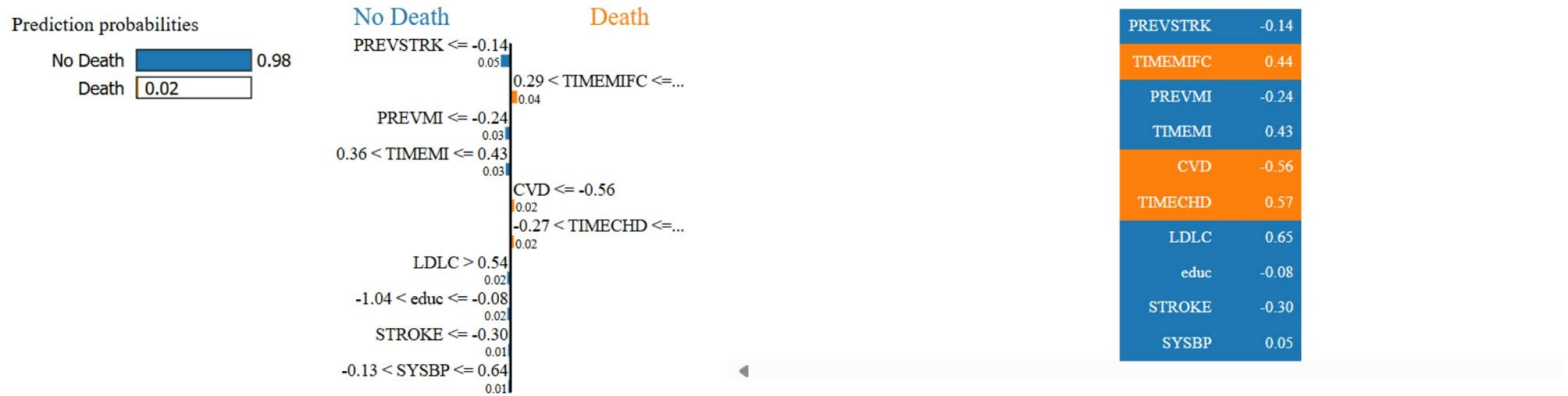
Local interpretable model-agnostic explanations (LIME) visualization for the support vector machine (SVM) model predicting survival (No Death) and mortality (Death) in the Framingham Heart Study. The figure shows predicted probabilities for each class and highlights the most influential features contributing to the prediction for an individual case. Blue bars indicate factors supporting survival, while orange bars indicate factors pushing the prediction toward mortality, enhancing interpretability of the black-box model’s decision process.

### LIME explanation for SVM predictions: death vs. survival

- This LIME visualization shows how individual features contribute to the SVM’s prediction that an individual will survive (No Death) or not survive (Death) based on Framingham Heart Study data (Fig. 21).
- *Prediction probability:* The SVM predicts a 98% chance of survival (No Death) and a 2% chance of death for this individual.
- These blue bars and negative signs (where appropriate) indicate these factors reduce risk of death.
- *Feature contributions:* Features pushing the prediction toward No Death (blue) -.
  - PREVSTRK (-0.14): No history of previous stroke supports survival.
  - PREVMI (-0.24): No previous myocardial infarction (heart attack) lowers risk.
  - TIMEMI (0.43): The time since last MI suggests lower immediate risk.
  - LDLC (0.65): LDL cholesterol level in the current range supports survival.
  - educ (-0.08): Higher education level contributes slightly toward survival.
  - STROKE (-0.30): No stroke history supports lower death risk.
  - SYSBP (0.05): Systolic blood pressure in this range favours survival.
- These orange bars are risk factors that pull the prediction toward death, but their combined effect is outweighed by the protective factors.
- *Features pushing the prediction toward Death (orange):*
  - TIMEMIFC (0.44): Recent myocardial infarction follow-up time contributes slightly toward risk.
  - CVD (-0.56): Presence of cardiovascular disease adds risk.
  - TIMECHD (0.57): Coronary heart disease duration slightly raises predicted risk.
- *Overall insight:*
  - The LIME output makes the black-box SVM interpretable by showing which clinical factors most strongly influence the prediction.
  - For this patient, strong protective factors (no prior stroke or MI, normal LDL, no recent stroke) dominate, explaining the high survival probability.
  - This helps clinicians and marketers understand which modifiable or non-modifiable risk factors matter, supporting personalized interventions.

LIME provides a transparent explanation for why this individual was predicted to survive. The LIME explanation for the SVM prediction highlights that factors such as absence of previous stroke (PREVSTRK), previous MI (PREVMI), and lower cardiovascular disease burden (CVD) significantly contribute in prediction. Conversely, factors like the presence of cardiovascular disease and coronary heart disease duration add risk but are outweighed by protective factors. This transparent explanation supports trust in AI-driven risk stratification for patient engagement and targeted intervention strategies.

## Results and comparison

This study utilized the Framingham Heart Study (FHS) dataset to develop and compare multiple predictive models for cardiovascular disease (CVD) risk assessment. The research employed a diverse suite of machine learning (ML) and deep learning (DL) algorithms—namely XGBoost, random forest, logistic regression, support vector machine (SVM), feed forward neural network (FFNN), and multi-layer perceptron (MLP)—alongside explainable AI (XAI) techniques such as SHAP and LIME to ensure transparency and interpretability in model predictions.

A. *Model performance evaluation*

The comparative performance analysis revealed that SVM outperformed all other models, achieving an exceptional accuracy of 96.65%, precision of 96.55%, recall of 87.50%, F1-score of 91.80%, and ROC-AUC of 99%. These results demonstrate SVM’s superior generalization and classification ability, making it the most effective model for cardiovascular risk prediction in this study.

In contrast, deep learning models such as the feed forward neural network (FFNN) and multi-layer perceptron (MLP) demonstrated moderate performance, with FFNN achieving a final test accuracy of 79.88%, and MLP achieving 95.98% accuracy and 98.06% AUC, indicating strong discriminative capability but slightly higher computational cost.

Among the traditional ML algorithms, Random forest and XGBoost displayed competitive performance, with accuracies around 79% and AUC scores above 0.82, while logistic regression performed slightly lower but offered faster optimization and simpler interpretability. Random forest’s marginally higher precision (74.22%) and stability can be attributed to its ensemble bagging nature, which effectively mitigates variance without extensive hyperparameter tuning.

The stacking ensemble model, which combined the outputs of XGBoost, random forest, and logistic regression using a meta-learner, further improved robustness and reduced individual model bias. However, SVM maintained superior predictive accuracy and calibration, reaffirming its suitability for structured biomedical data.

B. *Explainable AI (XAI) interpretability analysis*

To ensure the models’ decisions were transparent and clinically meaningful, Explainable AI tools were applied:

The SHAP Waterfall Plot revealed that AGE, ANYCHD (coronary heart disease), and CIGPDAY (daily cigarette consumption) were major contributors to death risk.

The SHAP Bee Swarm Plot demonstrated global feature impact, confirming AGE, PERIOD, ANYCHD, and SEX as consistently influential across samples.

The Feature Importance Chart quantified average SHAP values, identifying AGE (+ 0.87), PERIOD (+ 0.61), ANYCHD (+ 0.45), SEX (+ 0.28), and GLUCOSE (+ 0.22) as top predictors.

LIME (Local Interpretable Model-Agnostic Explanations) provided case-level validation, verifying that model predictions aligned with medically relevant features. This interpretability ensures ethical deployment and compliance in healthcare and pharmaceutical marketing contexts.

C. *Addressing class imbalance and overfitting*

The dataset exhibited class imbalance between survivors and non-survivors. Techniques such as feature correlation analysis and Bayesian hyperparameter optimization (via Hyper Opt) were employed to mitigate bias and avoid overfitting. Cross-validation confirmed consistent performance, while early stopping in neural networks prevented excessive training divergence.

D. *Implications for pharmaceutical marketing*

Integrating ML, DL, and XAI approaches in the FHS context has direct implications for data-driven pharmaceutical marketing.

The interpretable models provide actionable insights to identify high-risk patient groups, allowing marketers and healthcare providers to tailor interventions targeting modifiable risk factors such as smoking, cholesterol, and glucose control. The transparent, explainable nature of SHAP and LIME ensures ethical patient segmentation and supports precision-targeted outreach, aligning with regulatory expectations for AI transparency in healthcare.

By combining predictive accuracy with interpretability, this approach establishes a foundation for AI-driven marketing intelligence—transforming epidemiological insights into ethically responsible, precision marketing strategies.

The AI-driven models developed in this study demonstrated strong predictive performance across all key metrics, confirming their robustness and suitability for practical pharmaceutical applications. Advanced algorithms, including random forest, gradient boosting, and deep neural networks, successfully identified complex behavioral and clinical patterns. The integration of interpretable AI tools, such as SHAP and LIME, further strengthened the model’s transparency by revealing the underlying logic of pred. The resulting predictions offer a clear foundation for data-informed segmentation and targeted marketing communication. Distinct patient cohorts, identified based on treatment adherence, therapy response, or predicted disease risk, enable marketers to design focused and ethically aligned campaigns. For example, individuals in high-risk segments can be prioritized for therapeutic education, while lower-risk groups can receive preventive care information. This approach promotes a transition from broad product. In addition, the use of explainable model outputs enhances compliance with regulatory frameworks by ensuring that marketing strategies are guided by validated medical determinants rather than opaque algorithms. SHAP-based feature contributions reveal clinically relevant variables—such as comorbidities, prescription frequency, or demographic patterns—supporting transparent, evidence-based marketing practices. This interpretability fosters credibility among healthcare professionals and builds patient trust.

Furthermore, the model’s predictive capabilities can assist in forecasting market demand and optimizing campaign resource allocation. Probability-based risk projections can inform regional marketing priorities, while clustering outcomes may identify emerging therapeutic markets or underserved populations. This synergy between data analytics and marketing operations promotes adaptive, real-time decision-making that strengthens both commercial and social outcomes. In summary, these results confirm that technical model outputs are directly connected to strategic marketing functions. The integration of advanced analytics with domain expertise enables a transition from descriptive assessments to prescriptive, data-driven marketing systems that improve decision quality and maximize market effectiveness.

## Conclusion

This research validates the enduring value of the Framingham Heart Study (FHS) as a cornerstone dataset for cardiovascular prediction and decision-support modeling. By integrating machine learning, deep learning, and explainable AI, this study demonstrates how combining classical statistical insight with advanced computational techniques can produce highly accurate, transparent, and clinically relevant outcomes.

Among all tested models, Support vector machine (SVM) emerged as the most effective, delivering the highest predictive accuracy (96.65%) and robustness, outperforming other ML and DL models including random forest, XGBoost, logistic regression, FFNN, and MLP. The explainable AI frameworks (SHAP and LIME) provided vital interpretability, linking model predictions to medically meaningful factors such as AGE, PERIOD, ANYCHD, and CIGPDAY, thereby transforming predictive analytics into actionable clinical and marketing insights.

The study also highlights that while models achieved high accuracy, demographic and cohort limitations within the original FHS (primarily white, middle-class population) underscore the need for validation across diverse datasets to ensure fairness and generalizability. Future research should incorporate real-time digital health data, continuous monitoring systems, and multi-modal data fusion to further enhance predictive accuracy and model adaptability.

In a broader context, the conceptual bridge between epidemiological modeling (FHS) and marketing mix modeling (MMM) establishes a paradigm for evidence-based, interpretable, and strategic decision-making in pharmaceutical marketing. By leveraging the transparency and rigor of health analytics, marketers can design campaigns rooted in scientific reasoning and ethical AI deployment. This study provides a scalable framework that unites medical data science, machine learning, and explainable AI to improve clinical risk assessment, targeted healthcare delivery, and responsible pharmaceutical marketing—advancing the vision of a more transparent, data-driven healthcare ecosystem.

Ultimately, this study demonstrates a coherent connection between advanced machine learning models and their strategic use in pharmaceutical marketing. Beyond achieving technical precision, the proposed framework offers contextual intelligence for campaign planning, segmentation, and ethical audience engagement. Predictive and explainable outputs translate raw data into actionable insights, reinforcing the potential of AI to transform traditional marketing processes into intelligent, adaptive systems. By aligning model transparency with marketing accountability, the approach ensures that promotional strategies remain evidence-based, patient-focused, and compliant with healthcare standards. This balance of innovation and responsibility positions AI as a critical enabler of sustainable growth within the pharmaceutical industry. The research illustrates that integrating interpretable model outputs with marketing strategy transforms predictive analytics into a catalyst for informed, ethical, and effective pharmaceutical communication—bridging the gap between data science and patient-centered market impact.

## Future work

While the current research demonstrates valuable insights and predictive capabilities using the Framingham Heart Study dataset, several opportunities exist to expand and refine this work further. One important direction is to validate the developed models on more diverse and contemporary datasets, as the original Framingham cohort predominantly represents a white, middle-class population from Massachusetts. Incorporating data from multi-ethnic cohorts and different geographic regions will help ensure that the predictive models are generalizable and equitable across various demographic groups. Future studies should also explore integrating additional data sources, such as electronic health records, wearable device data, or genetic information, to enhance the granularity and predictive strength of the models. Another promising avenue is to adopt more advanced and interpretable explainable AI techniques to demystify the predictions of complex models like ensemble learners and deep neural networks. Improving transparency will help build trust among healthcare providers and patients. Longitudinal and temporal modeling could be expanded further to better capture the dynamic nature of risk factors, allowing researchers to study how lifestyle or medical interventions influence cardiovascular risk over time. Developing real-world applications, such as user-friendly clinical decision support tools or interactive dashboards, can translate these models into actionable insights for personalized care. Future work should also address potential biases by conducting thorough fairness audits and applying robust mitigation strategies to ensure ethical and unbiased predictions. Additionally, there is scope to benchmark new machine learning techniques, including federated learning or privacy-preserving AI, to respect data privacy while leveraging distributed datasets. Collaborating with clinicians and epidemiologists will remain vital to validate model outputs and ensure medical relevance. Regular updates to the models, incorporating new data from ongoing Framingham follow-ups or other studies, will keep predictions accurate and clinically meaningful. Lastly, fostering open research through the publication of well-documented code, reproducible pipelines, and transparent methodologies will encourage the broader scientific community to build upon this work and drive further innovation in cardiovascular disease prediction and prevention.

## Data Availability

Data supporting the findings of this study are available from the corresponding author upon reasonable request.
